# Current On-Skin Flexible Sensors, Materials, Manufacturing Approaches, and Study Trends for Health Monitoring: A Review

**DOI:** 10.1021/acssensors.3c02555

**Published:** 2024-02-23

**Authors:** Rodrigo G. Ferreira, Abílio P. Silva, João Nunes-Pereira

**Affiliations:** C-MAST, Centre for Mechanical and Aerospace Science and Technologies, Universidade da Beira Interior, Rua Marquês d’Ávila e Bolama, 6201-001 Covilhã, Portugal

**Keywords:** flexible sensors, active nanomaterials, polymer composites, manufacturing approaches, sensing performance, healthcare, physiological signals, electrochemistry, piezoresistivity, bioengineering

## Abstract

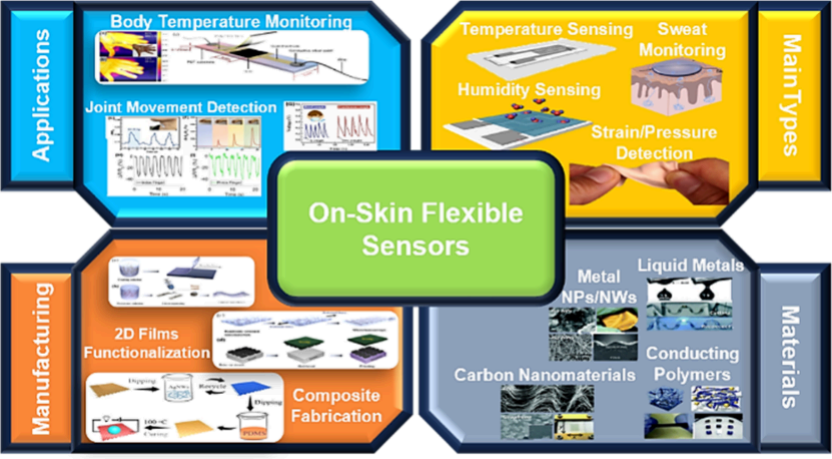

Due to an ever-increasing amount of the population focusing more on their personal health, thanks to rising living standards, there is a pressing need to improve personal healthcare devices. These devices presently require laborious, time-consuming, and convoluted procedures that heavily rely on cumbersome equipment, causing discomfort and pain for the patients during invasive methods such as sample-gathering, blood sampling, and other traditional benchtop techniques. The solution lies in the development of new flexible sensors with temperature, humidity, strain, pressure, and sweat detection and monitoring capabilities, mimicking some of the sensory capabilities of the skin. In this review, a comprehensive presentation of the themes regarding flexible sensors, chosen materials, manufacturing processes, and trends was made. It was concluded that carbon-based composite materials, along with graphene and its derivates, have garnered significant interest due to their electromechanical stability, extraordinary electrical conductivity, high specific surface area, variety, and relatively low cost.

With increasing longevity and quality of life, greater attention to health is required. At present, the standard methods of personal healthcare are mostly based on outdated techniques using bulky and heavy equipment, complicated and often difficult to access. Furthermore, patients frequently experience discomfort and even pain due to the invasive methods used to collect samples and data.^1,2^ Besides, wearable technology has evolved in recent years from wrist-worn fitness trackers to multipurpose sensors for real-time monitoring of physiological signals such as heart rate,^3^ blood oxygen levels,^4^ hydration,^5^ temperature,^6^ and sleep patterns.^7,8^ These new technologies make it easier to detect diseases in their early stages and to monitor their progression and treatment. As an emerging analytical tool, it can be placed on different parts of the body to collect biochemical and physiological parameters based on physical, chemical, and biological data through the skin. Tracking these indicators can greatly aid in the diagnosis, postoperative rehabilitation, and adjuvant treatment of patients with chronic diseases, especially those living in remote areas with limited access to healthcare.

On the other hand, since the 1970s, a wide range of applications for tactile sensors and electronic skins have been proposed in fields such as robotics, artificial intelligence, prosthetics, health monitoring technologies, and human–machine interfaces.^9^ Skin, one of the most amazing human organs, covers our entire body and has multiple nerve endings that can simultaneously sense, among others, pressure, temperature, and texture.

Recognizing this, rapid development in the research sector has focused on the use of flexible sensors that can detect pressure, temperature, humidity, and strain, with devices that theoretically almost mimic the sensory capabilities of the skin.^10^ They can be conformally attached to tissue surfaces in close proximity to the sampling site to record thermal, electrical, mechanical, and chemical changes, ensuring accuracy of detection and making them one of the most advanced health monitoring technologies. Wearable sensors must have high stability, specificity, and sensitivity to be effective as a tool for personal health care, and their sensing components must have excellent conductivity to convert various stimuli into electrical signals. All of these characteristics would ensure that wearable sensors would pick up variations from human motion to molecules and ions in body fluids.^1^

Wearable sensors are being extensively researched for their capacity to monitor personal health parameters continuously and accurately, allowing the domestic tracking of patients’ recovery after surgeries, avoiding long hospital stays, reducing costs, and minimizing exposure to nosocomial infections.^1,7^ For instance, pressure-sensitive electronics can help amputees and stroke victims regain sensory functions, and they can even be used to continuously monitor physiological health.^10^

As a result of these changes, there is a significant demand for flexible pressure sensors. Therefore, the development of wearable sensors has involved the use of functional materials, often including nanomaterials, which are used because of their high conductivity, fast electron transfer kinetics, and high aspect ratio.^1^

With the addition of nanomaterials these devices have outstanding electrical properties and are inherently flexible, providing both good tensile properties and sensitivity at the same time.^9^ Wearable sensors have improved significantly over the past decade, experiencing unprecedented market growth along with scientific advances in electronics microfabrication, flexible electronics, nanomaterials, wireless communication, artificial intelligence (AI), and communication technologies.^7,11^ Growing consumer demand and medical applications have also fueled market growth. In addition, there’s a trend toward miniaturization, which is critical to reducing the size of sensors and wearables, including wristwear, bodywear, and eyewear, which are increasingly influencing the healthcare and infotainment markets.^12^

Figure 1 shows that the market for wearable medical devices was worth $8 billion in 2020 and is expected to be worth $19 billion in 2025,^11^ with medical/fitness services leading the market for wearable sensor technologies, with demand growing rapidly amid the coronavirus (COVID-19) pandemic. Wearable technologies that monitor human body signals via flexible touch sensors have also received great attention due to their potential critical role in AI and the Internet of Things (IoT).^11^ Furthermore, due to remarkable changes in global Internet penetration, there is an increasing number of Internet-enabled smart devices in developing countries, especially in Southeast Asia and Oceania, which provides an opportunity for further growth.^12^

**Figure 1:**
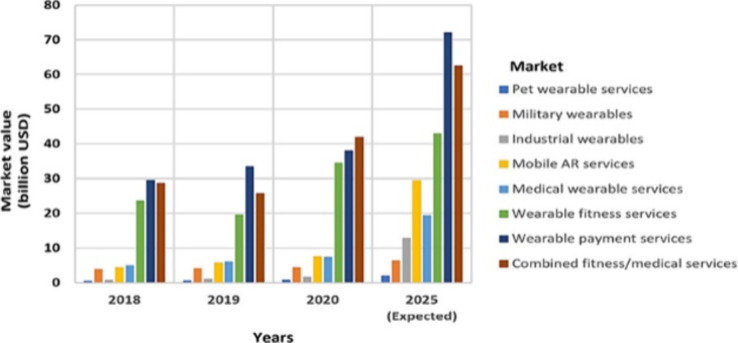
Market value evolution of various wearable services. Reprinted with permission from ref (11). Copyright 2022, MDPI.

However, looking at the challenges associated with progress in the field, despite all the developments made in recent years, many wearable sensors are still far from being reliably employed in long-term and continuous human activity monitoring applications, such as sweat sensors, while others are already achieving commercial success or are in later stages of technological maturation, such as temperature and strain sensors, respectively.^13−15^ Additionally, other technical challenges need to be faced before wearable devices can be used over the long term, including the lack of standards to date, limitations in microfabrication technology, stimulus-responsive material demands, and selection, and the debate between human-centered designs and truly personalized models, along with the lack of specifications and baselines for researching prototypes, consumer-grade, and clinical-grade systems, which is another obstacle to achieving effective validation and interoperability.^13−15^

Furthermore, most existing systems are being developed for the fitness industry rather than for older adults, seniors, and rehabilitation patients, which hinders the progress toward effective technology in these areas.^13^

A lack of common standards and interoperability issues prevent the efficient communication of sensor information and data, hindering the exchange of information between connected devices. Technical difficulties related to hardware and software are still prevalent today, including limited power reserves, small screens and displays, due to the compact size of the devices, or waterproofing issues due to sweat or washing causing damaging moisture in the electronics.^12^ In light of this, the potential of wearable technology still presents challenges that need to be overcome for the market to truly flourish.^12^ Looking at the challenges more closely, despite the trends and efforts mentioned, many wearable sensors are still far from being reliable for long-term and continuous human activity monitoring applications. Depending on the materials and their properties and function, further improvements are needed for each type of sensor in terms of form factor, mechanical and electrical properties, and energy efficiency.^16^ These concern user acceptance, data collection, validation, transmission, and battery life. However, other challenges must be improved for a long-term function, such as lack of standards, limitations of miniaturization, selection of electroactive materials and customization, i.e., human-centered technology,^13−15^ stretchability, response time, detection range, linearity, and hysteresis.^15^

One challenge is patient comfort in wearing a sensor that is in direct contact with the body or skin. To increase the popularity of these devices, they must be small and flexible, in order to be comfortable to wear, discrete and adaptable, without sacrificing their functionality.^15^ A greater commitment to ergonomic, accessible, intuitive, and simple interfaces and designs is necessary, as there is a high adaptability to different settings.^13,14^ On the other hand, customized models seem to meet the needs and constraints of specific individuals, although problems of interindividual variability, privacy issues and scale-related feasibility arise. Until now, the focus of most of these devices is capturing and promoting positive health behaviors while maintaining engagement.^13^

The functionality of these devices should also be considered, with a focus being on integrating sensing platforms into the human skin for physical and chemical target monitoring. These targets can be physical forces, such as strain, pressure, shear, torque, and vibration, physical–chemical parameters, including temperature and humidity, or biochemical parameters, including glucose, lactate, sodium, chlorine, and potassium.^17^ The most popular are based on changes in resistance, where physical stimuli cause shifts in the sensor’s resistance and conductance. Besides these approaches, other sensors operate on the variation of their capacitance or by piezoelectric and triboelectric mechanisms. While resistive and capacitive mechanisms require external power sources to function, the latter two generate their own power due to the manner their mechanisms are organized.^17^

Overall, given the potential of wearable sensors, research trends are looking toward low-cost, biocompatible, and eco-friendly materials with simple and cost-effective manufacturing processes and superior scalability.^16^ In light of the above barriers and challenges, further focus should be placed on materials development, exploiting specific properties and innovative manufacturing processes.

This work will address issues related to flexible on-skin sensors, materials, manufacturing approaches, and study trends, including material-related approaches that make up the devices, general concepts, operating principles, and their applications. Fabrication techniques will also be discussed, ending with a summary of the current state of the art.

## Components of Flexible Sensors

Over the past decade, flexible devices have advanced and shown immense potential not only in healthcare but also in robotics and biomedical engineering, where high sensitivity and accuracy are required along with flexibility and low cost. One application that stands out is flexible sensors for pressure sensing, which employ polymer-based flexible biocompatible materials, such as a biopolymer made from a blend of recycled polylactic acid (PLA) and wood, commercially known as Ecoflex, polyethylene terephthalate (PET), polyethylene naphthalate (PEN), silicone-based polymers, including polydimethylsiloxane (PDMS), and thin polymers such as parylene. These materials have good mechanical properties, a Young’s modulus similar to human skin, and high elongation limits, of up to 900% and 400%, for Ecoflex and PDMS, respectively.^18−20^

Wearable sensors can be placed on various parts of the body, including the head, eyes, chest, arms, and wrists, through flexible, textile-based, and epidermal-based approaches. As a result, physiological parameters and bodily fluids, such as saliva, urine, sweat, and blood,^21^ can be monitored and correlated to diagnose and even treat various diseases, where its drug delivery capabilities could be harnessed.^18^ A capability of mimicking the properties of human skin is fundamental with multifunctional sensing, while maintaining noninvasive, fast response to temperature and pressure, high resolution ratio, softness, low power consumption, and biocompatibility.^19,20,22^

### Stretchable Substrates

Substrates play an important role in the configuration of a flexible sensor. Many polymer films have been developed as substrates for flexible sensors and soft electrodes, such as polycarbonate (PC), thermoplastic polyurethane (TPU), polypyrrole (PPy), polyethylene terephthalate (PET), polyethylene naphthalate (PEN), and polyimide (PI),^23^ due to their excellent flexibility, thermal stability, good chemical resistance, and overall versatility, with some conductive polymers such as PPy being used in energy storage applications due to its excellent conductivity and electrochemical activity.^24,25^ In recent years, stretchability has also emerged as an interesting feature for flexible electronics materials, with PDMS being the simplest and most popular candidate as a stretchable substrate. Depending on the application, substrates such as polyurethane (PU) could be advantageous due to its proven compatibility with stretchable printed circuit boards (PCB), with common textile materials and electrospun elastic fibers.^26−28^

Although these substrates are not electrically conductive, they have acceptable gas permeability and biocompatibility and allow signal detection, conversion, and transmission through doping with conductive materials.^24^ Lightweight and thin substrates allow for a soft and curved surface that can be used continuously for long periods of time without causing discomfort to the user, while increasing conductivity helps to improve sensitivity.^24,29^ This must be supported by good adhesion, an important factor both for conformability to the skin and for attaching components to the substrate, which is typically achieved by surface treatments such as changing from hydrophobic to hydrophilic behavior through chemical functionalization, ultraviolet (UV) exposure, and oxygen plasma.^30^

Notwithstanding the widespread use of thin polymer films as substrates, several challenges remain: the fabrication and processing temperature of electrodes is limited by the lower thermal stability of polymers, typically below 200 °C, the time-dependent properties, long-term stability of polymer substrates can only be achieved through high permeability, and intrinsic poor adhesion of many of these polymers significantly limits applications and commercialization.^31^

Recently, hydrogels have been considered due to their biocompatibility, good mechanical properties, high content of water, self-healing properties, injectable abilities, and modulation during synthesis, advantageous assets in wound dressings,^32^ wearable ionic devices,^33^ flexible sensors, energy storing devices, soft robotics, and e-skins.^34^ Like the other candidates, conductive materials, such as carbon-based compounds and conductive polymers, can reinforce this substrate via polymerization and gelation, giving it high conductivity, boosting the already elastic properties of the hydrogel.^33^ Thus, unique capabilities can be achieved when varying the concentration of fillers, cross-linking state, and hydration,^33^ along with the ability to attach to the human skin and monitor physiological parameters in real-time, invaluable factors in the wearable sensor field.^33,34^

Devices would be able to monitor electrocardiograms, facial expression changes, and joint bending,^34^ while exhibiting antibacterial properties to prevent infections,^32^ self-healing and self-adhesive properties,^34^ and even monitoring wound pH values by converting hydrogel images into RGB signals.^32^

Despite this, low sensitivity plagues this material, due to poor interactions between rigid fillers and the soft matrix, spurring the urge to conceive new design and fabrication approaches to handle this issue.^33^

### Stretchable Conductors

Conductive materials are some of the most important elements in flexible electronics, as they connect all the components of the device. Optimal flexible conductors should exhibit high conductivity and strong robustness under mechanical deformation, and also the use of advanced materials and smart designs that greatly improve the wearability of the sensors.^24,29^ According to Lou et al.^29^ and Dickey,^35^ three strategies can be considered to produce stretchable conductors: intrinsically stretchable conductors, deterministic geometries, and composite materials.

Due to the limitations of traditional metallic and semiconductor-based sensors, polymer composites have emerged as the best alternative material, with the required stretchability for flexible applications.^25^ Stretchable conductors are usually introduced in the form of conductive fillers, such as nanoparticles (NPs), nanowires (NWs), graphene, conductive polymers, metals, and metal alloys, to functionalize stretchable polymer matrices, where the former provide the sensing features and the matrix acts as a flexible support structure. These allow for low percolation thresholds with high electrical properties and anisotropic packing, even for low filler concentrations. However, encapsulation and functionalization reduce the overall air permeability, breathability, and comfort of the composite sensor.^25,36^

With the goal of achieving excellent electromechanical stability at strains above 100%, several geometric designs have been reported, including wavy or serpentine-shaped structures,^7^ network patterns,^37^ 3D porous patterns, and crumpled structures,^38,39^ which impart stretchability to otherwise rigid metal films and conductive nanomaterials, while minimizing strain on the conductive materials and maintaining conductivity during reversible strain cycles.^1,29^

**Figure 2:**
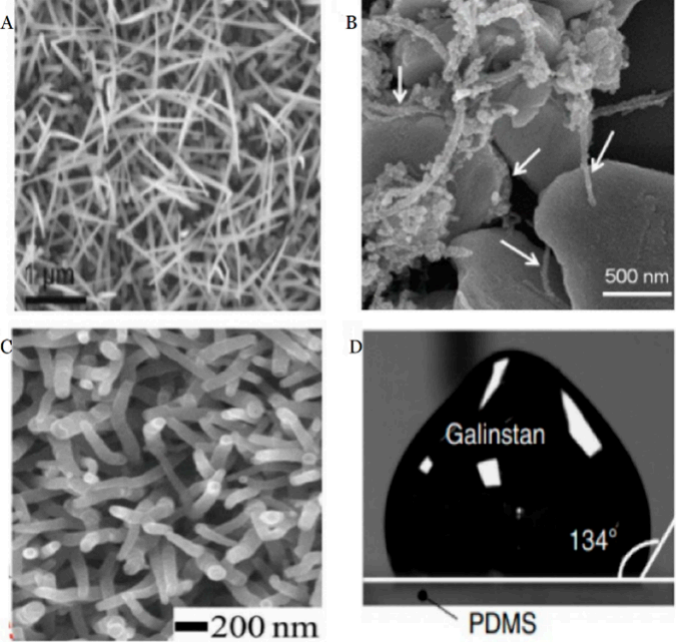
(A) Scanning electron microscopy (SEM) image of silver nanowires (scale bar 1 μm). (B) SEM image of Ag flakes anchored with Ag nanoparticles and multiwalled carbon nanotubes (MWCNT). (C) SEM image of PPy nanowires. (D) high surface tension and conductivity of Galistan. Reprinted with permission from ref (41). Copyright 2021, MDPI.

Material selection is an important factor in the improvement of response time and recovery characteristics, largely enabled by advances in manufacturing methods, which will be discussed further.^29^ In recent years, carbon nanotubes (CNTs), MXene, graphene, including graphene oxide (GO) and reduced graphene oxide (rGO), aluminum, silver or zinc oxide metal nanowires, particles, 2D semiconductors, organic polymers, and others have been regularly used as fillers. Carbon-based nanomaterials, such as carbon nanofibers (CNFs), CNTs, and graphene, have attracted attention due to their exemplary electromechanical properties and chemical stability, as well as well-known and explored synthesis techniques and scalability.^7,29,37,39^ This allows for high-capacity and low-cost production of fillers, which is in high demand in today’s market.^25,40^Figure 2 shows a scanning electron microscope image of silver nanowires, CNTs, PPy, and gallium–indium–tin alloy (Galinstan) used as stretchable electrically conductive nanomaterials.

## Main Types of Flexible Sensors

Broadly speaking, the electromechanical operating principle of the human brain functions by filtering and distinguishing important information from irrelevant information, prior to muscle responses. For that support, sensors collect data as a function of time, with subsequent transmission to the processor and activation of responses to the assigned condition.^42^ Today, research on physical sensing is responsible for most of the contributions to the field of wearable sensors, with sensors being woven and glued onto clothing or simply placed on the skin.^17,43^ These types of sensors are suitable for a wide range of outdoor and indoor applications, including body measurement, biomedicine, agriculture, and room and environmental monitoring, among many others.^44^ On the other hand, nonphysical sensors, such as sweat sensors, offer the greatest potential for continuous monitoring of biochemical parameters with the capability to detect biomarkers.^17^ Consequently, the data of interest give a huge amount of information, where selectivity will become critical.^42^

With this in mind, it is clear that material selection is critical in sensor design. Silver, despite its shortcomings, is used in the majority of designs, mainly for electrode patterning. Temperature sensors incorporate conductive polymers with optical transparency, plasticity and biocompatibility; humidity sensors mainly use the substrate as the sensing layer; mechanical strain and pressure sensors rely on polymer composites to achieve the required properties; sweat sensors function primarily through electrochemical sensing of biomarkers via biosensors.^44,45^Table 1 provides a summary of the sensing approaches with the positions and physiological relevance.

**Table 1:** General Summary of Physical and Nonphysical Sensing Parameters and Positions^17,42,44−46^

sensing parameter	sensing position	physiological relevance
temperature	skin	body temperature; blood flow; hydration
humidity	nose; mouth; skin;	respiration; dehydration; breathing patterns; respiratory conditions diagnosis
strain	hands; fingers; limbs; face; thorax; wrists; throat	body motion; phonation; facial expression; finger flexibility; hand gesture; respiration; pulse monitoring
pressure	hands; feet; wrists; neck; throat; hips; legs;	tactile sensing; hepatic sensing; diabetic foot; gait analysis; pulse monitoring; pressure ulcers; pressure control
sweat	skin	physicochemical health monitoring; dehydration; cystic fibrosis diagnosis; diabetes and kidney conditions monitoring

### Temperature Sensors

Body temperature is an elementary but vital parameter to monitor, as it provides an insight into a person’s physical condition, metabolism, and vital activity, since irregular variations in this parameter are indicators of certain diseases, that can cause high fever or hypothermia. Flexible temperature sensors allow these measurements to be taken at the level of skin, acting like a thermometer in which changes in electrical resistance reflect the body’s temperature and its variations, after being carefully calibrated. With a good-performing sensor, we could prevent diseases, perform screening and diagnosis, and revolutionize conventional medical treatments worldwide. To make this possible, the sensor must constantly monitor and regulate temperature to prevent abnormal physiological conditions, including infections that lead to hyperthermia.^17,42,47^

Wearable temperature sensors attached to the patient’s skin must be biocompatible and adaptable to the variable skin environment, especially with respect to sweating, to enable satisfactory long-term use. Currently, these sensors use a wide variety of materials such as nickel, silver, and copper nanoparticles and nanowires, CNTs, graphene, and conductive polymers as the thermal sensing elements.^45^

These sensors exploit the active material’s thermal properties, especially the thermal coefficient of resistance (TCR). The TCR is a valuable indicator of the sensitivity of the sensor and is defined as the relative change in resistance when the temperature changes by 1 °C. This is a critical parameter for RTD applications and is defined by eq 1(48)
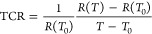
1where *R*(*T*) is the resistance at temperature *T* and *R*(*T*_0_) is the initial resistance measured at the initial temperature *T*_0_. Higher TCR values indicate higher accuracy. For human body applications, the sensing materials should have a high TCR on an interval between room temperature and 42 °C.^48^

The vast majority of these devices incorporate resistance temperature detectors (RTDs) or thermistors, either positive temperature coefficient (PTC) or negative temperature coefficient (NTC), with the main difference between the two being that the former has a faster response to temperature changes and better stability at higher values but has a smaller sensing range. The latter works best when a specific temperature, usually within 50 °C of ambient, needs to be maintained.^44^

Focusing on the RTD mechanism, it uses the dependence of the material’s electrical resistance on temperature to determine the TCR, where temperature increments cause an increase in resistance due to higher electron vibrations that prevent their free flow in the conductive material. A higher degree of accuracy, linearity, and faster response make RTD preferable to other options.^48^ Thus, the most basic configuration of a wearable temperature sensor can be achieved with a mesh-shaped metal circuit, whose resistance changes with temperature variation.^17^

**Figure 3:**
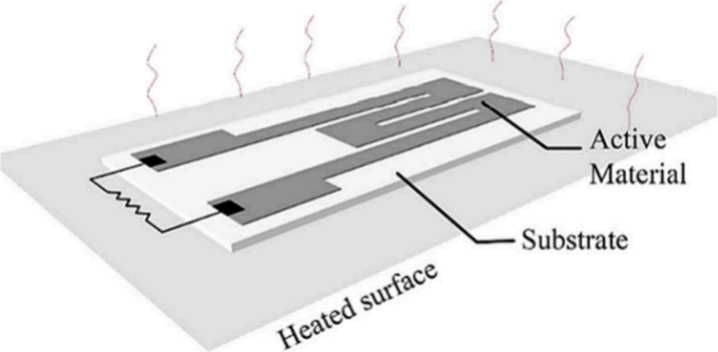
Typical setup for a flexible temperature sensor. Reprinted with permission from ref (44). Copyright 2021, MDPI.

Flexible substrates are the optimal choice for mounting the wearable sensor on the human skin, and the temperature sensing is usually performed on the back of the sensor, close to the skin.^44^ Due to the high demand for temperature detection in sensor systems, which makes the use of flexible sensors almost mandatory, functionalization with elastomers such as PDMS is very popular.^44,49^Figure 3 illustrates the general design of a temperature sensor. According to Bali et al.,^50^ Vuorinen et al.,^51^ and Honda et al.,^52^ composites containing poly(3,4-ethylenedioxythiophene), polystyrenesulfonate (PEDOT:PSS), and CNTs or graphene are temperature-sensitive organic materials common on temperature sensors with the possibility of being printed. The introduction of graphene as a temperature-sensing material is due to its excellent in-plane thermal conductivity. Furthermore, Ismail et al.^42^ claim that polymer/graphene composites are mechanically robust, not easily cracked or scratched during use, and highly sensitive to temperature. Silver (Ag)-based temperature sensors are also popular because silver is compatible with a wide range of substrates. Additionally, Kapton, a commercial polyimide (PI) film, is a good candidate for Ag-based sensors as its properties are already known and demonstrated.^53^

Nevertheless, several challenges remain in the development of temperature sensors. Ismail et al.^42^ reported that the majority of temperature sensors operate in the range of 10–80 °C. The lack of reports on temperature sensing above 120 °C is due to limitations in the thermal properties of the substrate, such as PDMS, PET, PU and even cellulose. In addition, the effect of mechanical stimuli on the performance of the sensor needs to be further investigated, as the resistance of a flexible temperature sensor can be affected by human-induced strain and stress, reducing its reliability.^54^ While the sensors must be accurate and reliable, skin-like conformability and stretchability are important features that cannot be ignored, where conformity to curved and irregular surfaces should always be considered.^54^ The solution is the use of highly deformable thermoplastic polymers such as PET, PDMS, Ecoflex, and PU. Structural arrangements can additionally improve the degree of stretchability, including serpentine, net-shaped, fractal, and noncoplanar designs, preventing the influence of body motion on the performance of the sensor, while improving sensitivity under fixed strains. The reliability of the sensor can also be monitored by subjecting it to cycles of temperature change and searching for variations in the resulting resistance/current peaks.^42,54^ Additional challenges include achieving humidity stability, as wearable sensors are inevitably exposed to ambient humidity, washability, where it is important to avoid degradation of the sensor after wash cycles, and breathability, where porosity, low thickness, and good substrate stretch must be achieved to allow the sensor to breathe when applied.^54^

### Humidity Sensors

Essential for life, humidity requirements vary dependent on the environments, but there is always a suitable range of humidity needed to promote survival and development.^48^ Also, it is an important industrial parameter in petrochemical processing, semiconductor manufacturing, aerospace, food and medical packaging and storage, and seed storage, where there is a growing need for more information on this parameter on cost, safety, comfort, and quality of human health.^55,48,56^ In particular, relative humidity (% RH) allows measuring the skin dryness, sweating, and breathing rate at a given temperature.^56,57^

These sensors work by absorbing water molecules either from the substrate or from the active films and diffusing them into the corresponding layer, causing changes in the electrical properties of the sensing layer.^44^ This is useful for monitoring tactile medical devices and the composition of air exhaled through the nose, which contains more moisture than inhaled air, allowing analysis of breathing patterns and diagnosis of respiratory diseases such as lung cancer, chronic obstructive pulmonary disease, and asthma.^17,45^

For low-complexity devices, a common approach is the deposition of a single active material on the substrate, followed by the exploitation of both materials’ physical properties for better sensing capabilities. Another approach would be functionalizing conductive electrodes on a substrate, measuring the response of the active layer/material, usually deposited on the electrodes.^44^

Regarding the operation, capacitive humidity sensors are the first choice because of their ease of manufacture, low power consumption, high sensitivity, quasi-linear response, and compatibility with modern technology.^46^ Thus, despite capacitance mechanisms being considered more complex, their remarkable performance compensates for the drawbacks.^44^ This type of sensor uses capacitance changes in the signal frequency and effective dielectric constant of ceramic or polymer dielectrics caused by losses or the presence of moisture, respectively. Their operation is made possible by the deposition of patterned electrodes on a substrate of variable thickness. Depending on the thickness of the film, sensors can be divided into thin-film types and thick-film types. Thin films allow for smaller sizes and greater sensitivity, while thicker films offer greater durability, cost-effectiveness, and reliable interfacing with other electronic circuitry.^46^

**Figure 4:**
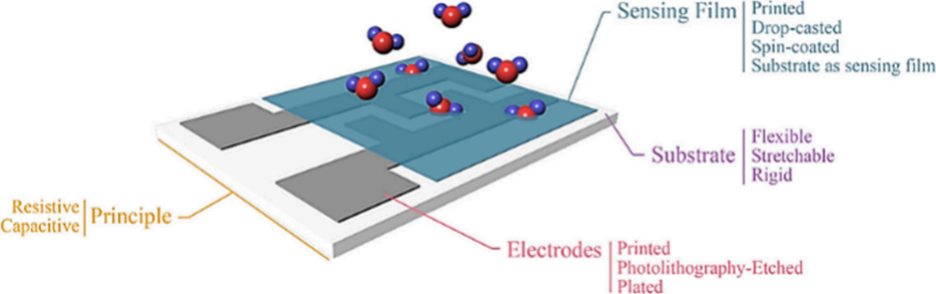
Possible humidity sensor setup. Reprinted with permission from ref (44). Copyright 2021, MDPI.

The ideal humidity sensor should therefore be easy to manufacture, low cost, mass-produced, reproducible, stable, highly sensitive, and linear, with low temperature drift and hysteresis and fast response and recovery times.^48^ Electronic biomaterials are attractive due to the high skin contact and the constant cycles of swelling and deswelling: i.e., the choice of substrate and reinforcement in order to obtain an active material is relevant, taking into account that humidity affects the conducting mechanisms.^42,44^ Silver^58^ and PEDOT:PSS^59^ can be used in humidity sensors with substrates of PI,^60^ PET,^61^ polyester,^62^ paper,^63^ or even ceramics^64^ and glass,^65^ depending on the manufacturing method, where the electrodes can be patterned and the sensing layer is printed on top. An array of possible configurations is shown in Figure 4.

In terms of challenges, one is the difficulty to desorb previously captured water molecules due to the nature of the active materials used, in particular multiwalled carbon nanotubes (MWCNTs), which offer high water absorption and a high surface area to volume ratio but lead to difficult desorption of water without external assistance.^60^ Thus, it is necessary to design and select material combinations that favor fast and repeatable absorption/desorption cycles, together with active materials that act as heaters to aid moisture desorption.^60^ Another challenge is manufacturing limitations due to temperature, such as paper, for example, the use of which is severely impacted. One way around this is to use drop-casting or spin-coating methods for material deposition.^66^

In summary, much work has been done to develop measurement techniques over a wide range of relative humidity (10–90% RH) due to the generally low accuracy requirements. However, detection in the low-humidity (<10% RH) and high-humidity (>90% RH) ranges is still challenging, especially at low-humidity conditions. This is due to limitations that also plague conventional humidity sensors, where the sensitive layer cannot absorb a large number of water molecules, making it very difficult to detect changes in the electrical signal, to the point where the sensor’s minimum detection threshold of the device is not reached by the water vapor concentration in the air.^46,48^

### Strain/Pressure Sensors

Force sensors are devices that can detect mechanical forces, such as stress, torque, stress, and pressure, followed by their conversion into electrical signals. Among them, pressure and strain sensors rise above the rest due to their ability to monitor physiological activity.^49^ Recent advances in microelectronics, nanotechnology, and fabrication methods have led to unprecedented miniaturization, high integration, and multifunctionality of wearable devices and electronics. This has created a demand for improved strain sensors with better functionality and flexibility, perfect for human health monitoring in skin applications.^67^ These stretchable and flexible sensors are the key to superior human motion monitoring, quantifying variations in electrical signals corresponding to physical deformations induced in the active material, useful tools for monitoring joint motion of fingers, elbows, knees and wrists, among other applications discussed below.^67^ Combined with fast data acquisition response times, high sensitivity, and good pressure/strain mapping, these sensors offer an interesting and promising prospect for research and future widespread use.^67^

Tracking a user’s habitual physical activity can provide useful information about, for example, walking and breathing patterns, posture, wrist pulse, sound vibrations, heartbeat, and hand movements.^68^ If abnormalities in these parameters are detected, such as sudden tremors and atypical gait patterns, it may be possible to observe precursors of diabetes or Parkinson’s and Alzheimer’s diseases, allowing for early diagnosis and treatment.^45,67^ Flexible strain sensors have opened up applications in all kinds of fields, including human–machine interfaces, rehabilitation enhancement, smart prosthetics, and sports training.^45^ Other fields can also benefit from these devices, including minimally invasive surgery and robotics, where exoskeletons could acquire sensing capabilities and perform even more complex tasks.^44,67^

Regarding the most popular operation principles, starting with the piezoresistive, this mechanism is defined by the variation of the internal resistance of the sensor when external stimuli are applied, followed by its conversion into an electrical output.^49,69^ In other words, each time the composite is deformed by mechanical forces, the contact area and disposition of the conductive materials within the matrix change, leading to a variation in conductance and, therefore, resistance.^49,69,70^ These resistance variations can be explained by three factors: changes in the contact resistance between different layers of materials, changes in the gaps between nanowires or nanoparticles, and changes in the geometry of the sensitive.^49^ Hence, piezoresistive sensors have received substantial attention thanks to their simple fabrication processes and structure, low power consumption and cost, and easy integration and signal acquisition, with a wide operating pressure range and fast response time, while possessing a large number of potential applications, especially medical diagnostic systems,^69^ human body motion monitoring, rehabilitation,^49^ and smart heartbeat monitoring for prevention of heart-related diseases.^70^

Capacitance-based strain/pressure sensors mainly rely on capacitance changes in the dielectric layer between two plates, converting applied pressure or strain deformations into capacitance changes. In the case of a parallel-plate capacitance sensor, the output value changes when the distance between the two plates is shortened by external forces.^70,71^ Thus, capacitance-based sensors are also widely used because of their high sensitivity over wide pressure ranges, low pressure detection thresholds, fast response time, durability, and applications in the wearable and health monitoring fields.^69^

Piezoelectric strain/pressure sensors are based on the piezoelectric effect, a mechanism widely used in sensor fabrication in which physical quantities such as acceleration, force, strain, and pressure, when applied in a given direction, are converted into measurable electrical quantities, due to the deformation of the anisotropic crystalline materials, leading to the polarization of internal dipoles and the generation of a potential difference between the two opposing surfaces of the crystal.^49,70,71^ Devices based on this sensing mechanism show great potential for dynamic pressure applications, benefiting from fast response times, low manufacturing costs, simple fabrication methods, and high sensitivity. Nevertheless, existing challenges revolve mainly around static pressure monitoring, since these materials generate voltage only when pressure is applied or removed,^49,69,70^ along with the existence of pyroelectric properties where the polarizations are massively affected by temperature, leading to output signal drifts.^70^

Depending on the installation and application, this type of sensor can also detect changes in pressure, bending, and touch, where the same sensor could detect both pressure and strain if deformation is applied to it.^44^ According to Gong et al.,^67^ many studies have been carried out on highly sensitive and even multifunctional sensors that can simultaneously detect strain, pressure, or touch. Since most commercial metal strain sensors limit the detection of human motion, researchers have turned to flexible sensors with high performance and stretchable properties. The sensor must be conformable enough to capture the full range of skin strain to perfectly reflect the physiological state.^17^ Most of the studies have been conducted using materials such as metal nanomaterials, graphene and GO, CNTs, and carbon black (CB).^67^

In terms of movement monitoring, there are two categories: large movements, which include finger, hand, and knee flexion, and small movements, which refer to subtle neck and chest activity during deglutition and breathing.^47^ Wang et al.^47^ reported that if sufficient reliability and validity can be achieved, activity monitoring has broad prospects in many clinical settings, not only for limb-related complications such as stroke and amputation rehabilitation but also for improved postoperative recovery from cardiac and pulmonary disease and for continuous monitoring of diabetic or chemotherapy patients.^47^ However, to achieve this, it is necessary to have high sensitivity, ultralow detection thresholds, fast response/recovery times, and a wide pressure range.^67^

**Figure 5:**
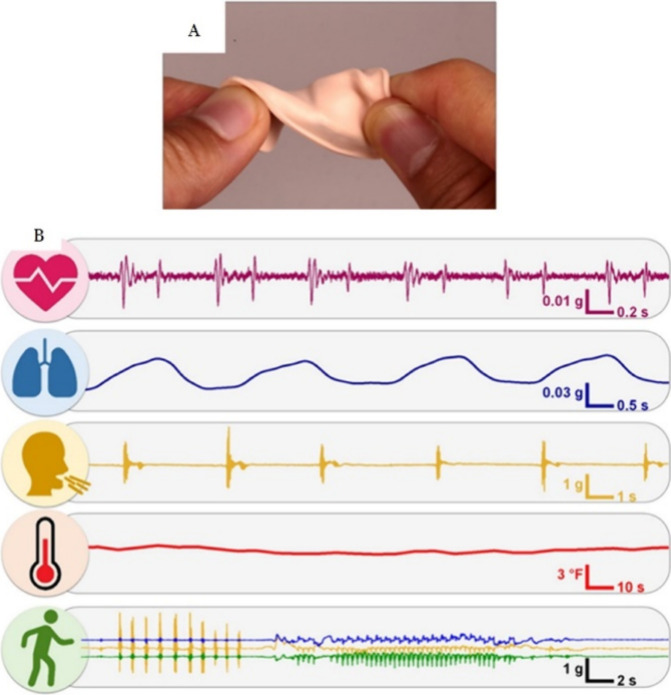
(A) Illustration of the mechanical robustness of a throat-worn pressure sensor. (B) Real-time monitoring of physiological parameters and human activity. Reprinted with permission from ref (42). Copyright 2022, Elsevier B.V.

Other applications include the detection of abnormal stress levels in the fingers due to hand disorders or unsatisfactory rehabilitation, interesting sport-related monitoring of elbow and knee joint movements, the detection of changes in stress induced by emotional facial expressions,^17^ which could help paralyzed patients to better interface with assistive devices, the study of breathing patterns and physiological processes through minute changes in the volume of body parts, and smart gloves,^43^ in which the softness or stiffness of objects can be reduced and perceived by the user when touched, benefiting the learning time of prostheses and the rehabilitation of amputees.^17,67^ In addition, pressure in other areas could be monitored to help manage pressure ulcers, assist bedridden and wheelchair-bound patients, and assess vocal cord disorders by attaching pressure and strain sensors on the skin covering the throat and evaluating the resulting vibrations.^17^ Human–machine interfaces could also be improved to better suit interaction with robots, where flexible pressure sensors would allow users to better receive the output signals.^67^Figure 5 illustrates the mechanical robustness of a flexible pressure sensor and its physiological signal response.

Despite recent advances, challenges related to response time, sensitivity, detection thresholds, and stability require new solutions.^67^ In practice, this can be adapted and even solved by the correct choice of active and passive materials, fabrication methods, and better system assembly, thus achieving an improved gauge factor (GF), eq 2, defined as the ratio of the relative change in electrical resistance to the mechanical strain^42^
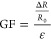
2where Δ*R* is the change in electrical resistance, *R*_0_ is the initial electrical resistance, and ε is the induced mechanical strain.^42^ In this sense, graphene-based strain sensors have gained popularity due to their favorable sensing properties, such as high sensitivity, low detection thresholds, low response time, and durability for long cycles of use. This type of sensor can detect all types of physiological movement, including vocal-cord-induced vibrations,^72^ with a stretchability of at least 100% and a GF in the 5–100 range.^42^

Skin-like pressure sensors face another difficulty in the form of airtight membranes with gas permeability, which cause skin irritation and allergies after prolonged use. To solve this problem, breathable layers with sensing capabilities are crucial for this type of electronics.^67^

Focusing specifically on piezoresistive sensing, commercial strain sensors are directionally fixed and able to measure only very small strains, of less than 5%. Therefore, these approaches are not suitable for flexible and wearable sensors, since multidirectional, high sensitivity, wide pressure range, and even multifunctional characteristics are most often required. Taking this into account, novel sensing elements based on nanomaterials will be required to achieve these goals.^71^ In turn, embedding conductive materials in elastomer matrices with porous structures is a great way to achieve two- and three-dimensional conductive networks capable of providing high performance for piezoresistive sensors, with potentially ultrahigh sensitivity to applied strain or pressure.^49^

### Sweat Sensors

Sweat is rich in chemical information, containing biomarkers that reflect the biomolecular state and fitness level of the individual.^45,73^ During perspiration, the sweat glands of the average adult human produce between 500 and 700 mL of hypotonic fluid per day. This is the body’s primary means of thermoregulation, and a number of biomolecules, hormones, proteins, amino acids, peptides, ions, and metabolites are excreted during this process, most notably lactate, glucose, uric acid (UA), ascorbic acid, and cortisol. Depending on the concentrations of these elements, conclusions can be drawn about the biomolecular state of the body, including continuous monitoring of physical health through the concentration of metabolites such as lactate, or by looking at pH levels and the concentration of Na^+^, K^+^, and Cl^–^ ions, which are very similar to the blood and can reflect its health through a less invasive and uncomfortable procedure.^45,74,75^

The ease of access makes sweat a particularly useful biofluid because it provides information about diet, drug use, health status, and dehydration, among others.^45,67^ For example, an increase in chloride ion concentration normally indicates dehydration,^76^ while abnormal losses of sodium ions may indicate cystic fibrosis or a related genetic disorder.^45,74,77^ Correlations between sweat and blood glucose levels can aid in diabetes diagnosis and monitoring, while lactate levels can detect ischemia.^45,74^ On the other hand, high levels of urea in sweat are also associated with impending kidney failure.^45,74^

**Figure 6:**
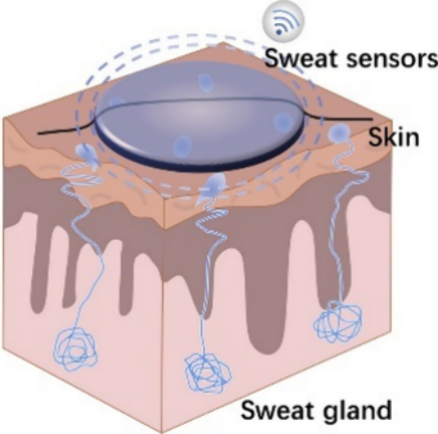
Structure of a sweat gland (cross-section view), with a wearable sensor placed on the skin’s surface. The produced sweat is absorbed by the hydrogel-based sensor and its swelling recorded and analyzed for real-time sweat volume monitoring. Reprinted in part with permission from ref (82). Copyright 2021, Elsevier B.V.

Sweat analysis has largely been achieved through novel molecular recognition methods, integrated hardware/software systems, nano/micro manufacturing approaches,^78^ and multiple analytical approaches that include electrochemical, fluorescence, surface-enhanced Raman scattering (SERS), and colorimetric sensing methods with the ability to diagnose complications in the early stages and opening the doors to personalized treatment.^34,79^Figure 6 shows a superabsorbent hydrogel-based wearable sensor for real-time sweat volume monitoring, one of the possible applications of these devices. Among these, electrochemical sensing is a well-established and advantageous technique for implementation in sweat-sensing devices due to the high sensitivity, low cost, and ease of miniaturization.^74,80^ These sensors operate by transducing the analyte concentration into electrical signals.^78^ Thus, the biological component chosen for the biosensor’s recognition system must be dependent on the desired analyte, while being able to output its concentration as an identifiable and measurable physicochemical signal. The transducer must be selected depending on the bioreceptor and measurement technique. Currently, the most crucial analytes for this method are electrolytes and metabolites, while the most used detection methods are enzymatic amperometric and potentiometric ion-selective electrode sensors.^74^

Starting with amperometry, this is a dynamic approach to interfacial sensing methods in which direct or indirect responses can be observed in the presence of a specific analyte on an electrode surface, resulting in the generation of a measurable electrical signal disturbance. The electron transfer between the electrode and the analyte, during its oxidation or reduction, generates a current proportional to the concentration of the electroactive product.^80^ These sensors employ three electrodes, the working electrode, the reference electrode, and the counter electrode, all deposited on a flexible substrate. The reference electrode has a known and stable electrical potential and is used to determine the electrical potential of the working electrode.^74^

Ion-selective electrode sensors, or ISEs, are transducers capable of converting specific ion activities into readable electrical signals.^74^ Traditional ISEs employ liquid contacts, also known as inner filling solutions, that separate the sensing membrane from the inner reference element. Using the Nernst equation, the logarithm of the ion activity can be correlated to the generated voltage, which achieves the target selectivity through direct potentiometry. In ISE sensors, the ISE acts as the working electrode and a reference electrode is required, similar to the amperometric sensors mentioned above.^74^

Despite this, ISE sensors also require delicate fabrication methods and maintenance processes, which is a challenge for miniaturization. Furthermore, it has been observed that the potential at the interface between the membrane and the metal contact can become unstable due to the transition from ionic to electronic conduction in the membrane. In this sense, the formation of a water layer in the polymeric membrane is possible due to the uptake and diffusion of water molecules, leading to the failure of the whole device. Therefore, more research is needed on the ion-to-electron transduction mechanisms and the layers between the sensing membrane and the electron-conducting substrate to achieve stable responses from the sensor.^80^

Despite the advantages of these mechanisms, much work still needs to be done to develop electrochemical sensors that remain stable over long periods of time. This behavior is exacerbated by the unstable nature of enzymes when immobilized, which further affects sensor sensitivity and stability. Regardless of the targeted enzyme and immobilization approach, electrochemical sensors lack full reproducibility, factors that make large-scale production and scalability a laborious endeavor.^81^

Other methods have also attracted interest, where fluorescent and colorimetric-based sensors provide visual cues, via observation of color/fluorescence/absorbance, related to analyte content.^78^ The colorimetric mechanism is the most practical of the two thanks to its cost-effectiveness, simplicity, versatility in various environments, and detection with the naked eye.^34^ Due to the continuous perspiration generated by the human body, sweat can be periodically collected in superhydrophilic microwells that allow reaction with the colorimetric reagents, producing optical signals that change depending on the analyte concentration.^34,79^ These RGB signal changes can then be analyzed by a smartphone and converted into easily readable information accessible to the user.^34,79^

SERS is another method capable of effectively enhancing Raman signals through plasmon-enhanced excitation and scattering phenomena and has attracted considerable attention in the biomedical field. Despite this, and some prototypes being developed, by not relying on microfluidic systems, these porous substrates exhibit unstable structures and low linearity and refresh times.^78^

They also need to be adaptable (elastic) to deformation of the skin as flexible sensors, while new advances in sweat collection methods are crucial.^45,46,74^ Good analytical performance in healthcare and fitness depends on the composition, layer configuration, and wearability of the sensor. According to Gong et al.,^67^ carbon black (CB) has some valuable advantages, including excellent electrochemical properties, easy preparation of stable dispersants, and simple fabrication. Thus, not only are biomaterials used in sweat sensors but also nanostructured metal oxides, which play a good role in electrochemical sensing, where printing or functionalization of these materials on flexible substrates could lead to interesting new prospects for monitoring.^67^

Although sweat provides a good base of physiological information, there are still barriers to its proper use: namely, there is a lack of correlation between sweat and blood analytical information. Although the measured analytes are well documented, such as steroid hormones and drugs, the complex composition of this biofluid makes the detection of multiple biomarkers with a single wearable device a major challenge for real-time signal processing.^45,69,74^ Another major challenge is the lack of suitable active materials with adequate flexibility, elasticity, and transparency and acceptable mechanical properties. In reality, conventional electrochemical sensors are rigid and heavy and depend on bulky electronic support systems that hardly support cyclic multiaxial mechanical deformations and can be miniaturized; therefore, they are almost incompatible with wearable applications.^45^

Finally, considering that the majority of sweat is produced by eccrine sweat glands, and that sweat is transported to the skin surface by dermal ducts, it can be concluded that the required ultrasensitivity and selectivity for some analytes may be compromised by the sheer number of cellular barriers that must be crossed, even with thorough sweat collection systems. This is particularly true for larger biomarkers, where the degree of filtration by tighter junctions is increased, resulting in greater dilution. The most prominent example of this is glucose, which is transported by paracellular pathways and is approximately 100 times more dilute than glucose found in blood plasma or interstitial fluid, presenting an even greater challenge to wearable sensing.^74^ To counteract this, sensors for physiological data collection must be in close contact with the skin, allowing in situ sweat collection and analysis, with wireless signal transmission via Bluetooth and NFC, or other simplified protocol.^45,74^

### Other Sensors

With the rise of smart devices in our daily lives, wearable electronics appear as a great candidate to meet the needs of the population, with the added capability of skin conformability, flexibility, and stretchability, rendering them a great invention and breakthrough in the healthcare field.^83,84^ At the same time, textiles are essential materials and are a part of human nature, with cotton, wool, and silk, among others, being natural forms that have been crucial to civilizations throughout history. When unavailable, synthetic forms, such as polyamide and polyester, are also able to create fabrics with wearability, reusability, breathability, washability, fashionability, and durability, or even to enhance the functionality of more traditional materials.^83,84^

Nowadays, smart textiles, materials that interact with the surrounding environment and serve multiple purposes, are being developed to harness the potential of wireless networks, artificial intelligence, and big data in novel personalized theragnostic and point-of-care approaches, using both physical and chemical solutions.^83,84^ This implementation can lead to significant cost savings along with an optimized social welfare system, depending on the level of complexity of the devices, ranging from passive smart textiles that sense changes in the environment, to active textiles that can detect and respond upon external stimuli, ending with smart textiles that have the means to monitor, react, and adjust their properties according to the situation and its stimuli, while maintaining biocompatibility and humidity resistance.^83,84^

In these applications, commonly used reinforcing materials are 2D nanomaterials, such as graphene, which due to their physicochemical properties are able to boost carrier mobility to outstanding levels, making them suitable candidates for these systems.^83^ On the other hand, other compounds could be used as electrodes or energy generators, including metals, electroactive polymers, and other carbon-based materials, either to facilitate data acquisition or to power the entire theragnostic system.^84^ Textile-based sensors could also employ optical approaches to detect and produce light in the presence of target metabolites, blood pressure, and heart rate, among others, aiding the biomechanical component of the device.^84^

Devices that integrate these materials would be able to convert and relay physiological inputs to clinical staff, taking the healthcare field into a new level of personalization, prevention, and prediction, and enabling the development of novel devices such as textile hearing aids, prosthetics, sign-to-speech smart gloves, textiles with exoskeletal support, and electrical stimulation for tissue rehabilitation, pain relief, and wound healing.^84^ However, when considering power supplies for textile-based systems, despite the advances made toward flexibility of textile platforms, some approaches can compromise the form factor, freedom of movement, and comfort of the device, while adding weight.^84,85^

Another group of devices that have been attracting great attention are electronic tattoos, also known as e-tattoos, noninvasive epidermal electronics placed in close proximity to the skin with the ability to monitor physiological parameters in real time and interface with users.^85,86^ Due to the developments regarding biocompatible materials and sensor technology, previously unattainable biological signals can now be monitored while maintaining minimal contact with the skin for short periods of time, up to 2 weeks.^85^

Therefore, these advanced electronics will eventually replace some of the more traditional systems due to their potential to be inexpensive, conformable to the skin, comfortable, and even potentially multifunctional.^86^ These properties can be achieved by using thin devices with a low Young’s modulus and high adhesion matrices, along with conductive materials with good electrical properties and transparency.^86^ Achieving this would mean that e-tattoos would be able to monitor biological signals directly through the epidermis, without being perceived during wear or causing foreign body sensation, while also opening the doors to advances in the areas of drug delivery systems, where the timely delivery of drugs across the dermis to a target area upon detection of a trigger would allow for optimized diagnosis and treatment of a wide range of conditions.^85,86^ Other applications include electrodes in neuro-interfaces, recording electro-oculograms (EOG),^87^ monitoring heart and brain activity through electrocardiograms (ECG)^88^ and electroencephalograms (EEG),^89^ among other biopotentials such as body temperature, all thanks to the inconspicuous nature of the e-tattoo.^90^

Some manufacturing approaches aiming for breathable e-tattoos include phase separation, electrostatic spinning, and template-based methods, while others, such as spin-coating, are effective in producing ultrathin structures but lack the ability to achieve breathability, a challenge that, while persistent, will impact the ability to meet the ever-growing demand for these devices.^86^ Some methods require significantly higher costs and equipment requirements, while exhibiting low efficiencies and difficult to control processes, so the need for highly efficient and affordable approaches remains.^86^ Other challenges include the accumulation of sweat leading to degraded signal quality, exacerbated in nonbreathable epidermal electronics, skin inflammation,^86^ potentially limited biological signal detection due to minimal direct skin contact of the active elements,^85^ and the overall need to address the dynamic and diverse nature of the real world, where the scope of continuous monitoring devices must rapidly expand to meet the demands of more personalized therapies.^84−86^

### Commercial Challenges and Strategies

Despite significant advances, several challenges must be overcome before wearable sensors can truly be widely adopted by the commercial market. This can only be achieved through continued research that leads to more affordable and comfortable devices, regulatory approval, and a change in the way healthcare is viewed.^91^ Some challenges include the employed materials, where issues of low mechanical strength, durability, and thermal stability combined with high viscosity can lead to difficulties in producing devices with the required consistency, as well as low long-term stability under physical deformation, loss of repeatability and electrical properties with repeated use, and reduced sensitivity.^91,92^ However, these complications affect natural polymers significantly more than synthetic forms. The solution to these problems could be to blend the two forms, if they are compatible, and cross-link them by physical or chemical processes, resulting in new composites with optimal properties.^91^ Unfortunately, many natural polymers are available in small quantities, which, combined with difficult processing and low production volumes, leads to very expensive manufacturing approaches, despite the need for wearable sensors that require biocompatibility and comfort.^91^

Another challenge is to maintain reliable sensing performance under everyday conditions, especially in the case of electrochemical sensors, which must withstand tensile and strain deformation to obtain good samples, on top of compensating for temperature and humidity variations and electrochemical properties that are pH dependent.^91,92^ This is also related to the problems that can arise when trying to store these devices for long periods of time, where low storage stability affects their biorecognition capabilities, reducing the practicality of biosensors, along with the release of carcinogenic compounds, microplastics, and other chemicals when the sensors are disposed of, negatively impacting the environment with resource depletion, pollution, and biohazardous waste, as well as human and ecosystem toxicity due to contamination of the food chain.^92^

Other risks include the possible contamination of excretions such as saliva, sweat, and tears, as these substances have a much higher risk of contamination than blood samples, exacerbated by the tendency of some hydrogel and elastomer-based materials to provide a compatible environment for bacterial growth, along with the presence of food residues, dust, and cosmetics. Hence, selectivity must be increased to specifically target these interferences.^92^ Calibrating the devices to minimize artifacts is also a challenge, as separating noise from the biological signal is difficult due to the presence of electromagnetic interference, physiological signal cross-detection, and motion artifacts.^91^

Lastly, the large volume of data generated by these collection processes can lead to analysis and management issues, creating difficulties in transmitting key data between patients and clinical staff via wireless approaches, compounded by privacy issues and a general lack of standards between research teams and manufacturers.^91,93^

With this in mind, several strategies could be considered to achieve commercial success, including the development of interoperability standards, making wearable sensors an affordable option for most people, using environmentally friendly and cost-effective solvents in the production of biopolymers, reducing background noise, improving long-term stability, durability, reusability, reproducibility, and pH stability of both physical and electrochemical sensors, along with storage stability, which can be enhanced by the use of appropriate gels when encapsulated.^91,92^

## Materials in Flexible Sensors

Efforts have been made in nanotechnology and materials engineering to design sensors with greater potential for miniaturization, conformability, and skin adherence. This is made possible by controlling the nano- and microscale morphologies of inorganic, organic, and even hybrid materials, which allows for the improvement of properties, such as lighter weight, flexibility, and ultrathinness, improving the overall performance and functionality of the sensor.^94,95^ Due to their inherent mechanical stretchability, these materials can be manufactured into many types of membranes for on-skin applications, which are used in wearable sensors. These can also provide an important structural element in the signal transmission mechanisms.^24^ Despite this, the challenge remains to develop skin-attachable healthcare devices that incorporate flexible and stretchable interconnects, multifunctional sensors, and wireless communication, while maintaining a viable power supply.^94^

The main focus of the essential components of a flexible electronic device must be the substrate, the active layer, and its interface. Regarding the active layer, both organic and inorganic options have been considered. While organic materials have gathered attention mainly because of their flexibility, the latter have good physicochemical properties, chemical durability, and mechanical strength, along with high electron mobility.^95^ On the other hand, silver nanoparticle (AgNP) composites are also functionalized as the active conductive layer in many flexible substrates, where the interface is also an important issue to be discussed.^95^

For wearable sensors based on polymer composites, carbon base materials, such as graphene, CNF, CNT, and metal fillers, like silver nanowires and nanoparticles, are the most used materials for the active layer. Metal fillers tend to have relatively high cost, poor surface modification, nonlinearity, and poor acid and alkali resistance, which limit their durability and application in the physiological signal monitoring field.^96,24^

### Substrates

Most substrates only have the function of supporting the stresses by the entire device, such as bending and stretching, where the main requirement is a low Young’s modulus and high resistance to cracking. Therefore, the first choices for the substrate or matrix, in the case of composites, are inherently stretchable materials, especially elastomers, due to their large-scale deformation, their ability to withstand dynamic strains of more than 100%, and their durability, with unchanged properties after thousands of cycles.^97^ Common substrate materials are polyurethane- or silicone-based.^98^ Currently, there is a focus on PDMS as a substrate for the development of skin-like stretchable sensors, using surface modifications as a means to control the adhesion and interactions between the substrate and the conductive materials.^99^ Some thermoplastic elastomers, such as TPU, are also used as the matrix/substrate for systems that require higher stretchability than PDMS, typically for 120–160% strains.^97^ However, the sensitivity of these materials can be affected by temperature, fatigue, and creep.^100^

Thermoplastics and thermosets, including polyvinylidene fluoride (PVDF), polypropylene (PP), parylene, and epoxy, have also been proposed for high-strength applications, some of which, such as parylene, are known for their biocompatibility, chemical inertness, and low permeability to moisture and are already widely used on a large scale in implantable and microelectromechanical systems (MEMS) devices.^101^ Nevertheless, after functionalization with conductive fillers, toughness and ductility are usually sacrificed, resulting in smaller elastic regions and narrower strain sensing ranges. Thus, the application of too much strain causes irreversible changes in the substrate/matrix and in the active layer. Thermoplastics are mainly used in structural health monitoring applications.^102,103^ Paper and silk fibers, as well as low-cost and recyclable materials, have also been used for their flexibility, comfort, and biocompatibility.^101,104^ These materials support stackable architectures and provide high conformability and deformability, with applications ranging from temperature monitoring to heart rate, blood pressure, and skin hydration monitoring.^101^

To be successful, acceptable sensitivity must be achieved over a wide strain range. According to Kanoun et al.,^105^ increasing the Young’s modulus of a material leads to enhanced sensing performance because mechanically induced hysteresis leads to nonlinearity and imperfect sensing performance with long response and recovery times; thus, low-hysteretic substrates are preferable for strain sensor fabrication.^98^ However, according to Bunea et al.,^101^ reducing the Young’s modulus of the substrate can lead to greater comfort when using devices on the skin, with special attention to substrates made of materials with flexibility and native or induced stability, thus allowing conformal integration of the necessary electronic components on the skin, avoiding mechanical degradation of the sensor during operation.^101^

### Conductive Fillers

**Figure 7:**
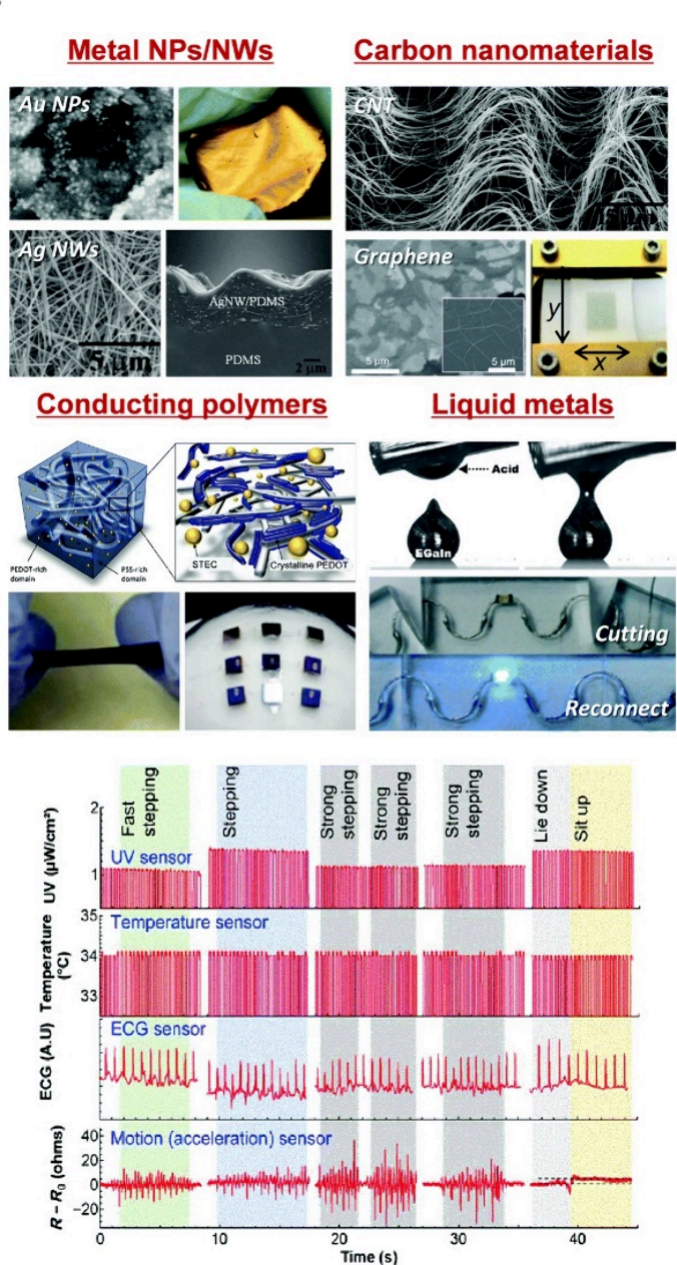
Stretchable hybrid materials employed as active materials in composite wearable sensors, along with wearable multimodal health monitoring graphs for the monitoring of various physiological signals. Reprinted with permission from ref (94). Copyright 2018, The Royal Society of Chemistry.

For flexible sensing applications, the development of stretchable conductors with high performance, stretchability, and stable electrical conductivity is essential to create better electrodes for sensing elements, wireless antennas, and interconnect components for the new generation of wearable devices.^94^ Typical materials used as conductive fillers are illustrated in Figure 7. Considering the majority of sensing mechanisms, it is possible to functionalize an insulated polymer with conductive fillers, such as carbon black, CNT, metal nanoparticles, graphene, and MXenes, to obtain a composite material with an internal conductive network.^25^

According to Ha et al.,^94^ some of the methods used to obtain the conductive material are simple deposition of the conductive nanomaterials on the surface of the polymer substrate and embedding conductive nanomaterials in the stretchable polymers, through multiple layers. This way, the goal is fabrication of composite materials where the polymer matrix provides flexibility and stretchability, while the nanofillers confer the desired electrical conductivity to the material.

Over the years, metal nanoparticles have been considered unsuitable as active fillers for stretchable matrices, due to the high values of percolation concentration values required to create a fully conductive network within the matrix. Besides that, the contact resistance observed at a large number of interparticle junctions is also a major problem. Currently, attention has turned to metal nanowires,^106^ CNTs,^107^ and graphene,^108^ which allow for highly sensitive conductive networks with unique morphologies, at low percolation, while being able to achieve and maintain electrical conductivity throughout its operation.^94^

The embedding of conductive layers instead of the direct surface deposition of conductive materials provides additional robustness to the structure without risk of delamination during load/unload cycles. Thus, hybridized graphene and CNT are a very popular choice; as an example graphene 2D nanostructures have an excellent electrical conduction and mechanical strength for functionalization of flexible substrates, resulting in reliable and continuous electrical conductivity along with improved mechanical properties, achieved in a lower concentration percolation.^94^ Metal conductive fillers, commonly nanoflakes and nanowires, are 1D nanoparticles used for the manufacture of flexible composites. Unlike carbon-based materials, whose properties are influenced by the manufacturing process and are subject to agglomeration, metal-based nanowires offer high conductivity, large surface area, and high aspect ratio. However, they are also known for their nonlinearity, poor adhesion to the substrate, and tendency to oxidize with prolonged exposure, factors that sacrifice device lifetime.^25^ Furthermore, inducing high strains can lead to irreversible gaps and cracks between nanoparticles, resulting in devices with a limited strain range.^98^ Metallic nanowires are mainly made from silver, copper, gold, and platinum, with silver currently being the most widely used.^25^

Graphene, consisting of a single layer of carbon atoms arranged in tightly bonded hexagonal rings, is one of the most widely used nanomaterials because of its superior flexibility, excellent electromechanical properties, biocompatibility, light weight, large specific surface area, and tunable 2D structure, along with an optical transmittance of ∼98%^109^ and a Young’s modulus of around 1000 GPa, perfect for flexible sensor development.^110^ Graphene can be used in sensors in multiple dimensions, such as 1D fibers, 2D films, and 3D monoliths. Other forms of regular graphene, such as GO, rGO, graphene ribbons, graphene sheets, and nanoparticles, can be used as building blocks for other materials needed to achieve higher performance. Among these, GO and rGO stand out, with GO exhibiting semiconducting behavior, while rGO is highly conductive, almost at the same level as pristine graphene.^25^ 2D graphene films are popular mainly due to their high flexibility, light weight, conformability, and simple fabrication processes, which can achieve a large contact area with the human skin and thus capture physiological signals more reliably. Graphene films can be obtained from GO solutions by vacuum-filtration, spin-coating, spray-coating, wet-spinning, dip-coating, and self-assembly methods, followed by reduction by physical or chemical methods to take advantage of the high conductivity of rGO.^25^ Lastly, an emerging research topic with the potential to replace 2D nanostructures regards 3D graphene structures consisting of monoliths, which are foams with high porosity, large surface area, and good structural stability and can be prepared by electrospinning, 3D printing, or template-directed methods.^111^

Carbon nanotubes (CNTs) are widely used carbon allotrope nanofillers that consist of a graphene sheet forming a cylindrical shell, with lengths of up to hundreds of nanometers and diameters of a few nanometers, resulting in high aspect ratios and are often considered a 1D structure. CNTs can further be divided into two categories based on the number of carbon atom layers, namely single-walled CNTs (SWCNTs) and multiwalled CNTs (MWCNTs). SWCNTs are generally preferred due to their higher flexibility, thermal conductivity, mechanical strength, and aspect ratio.^25^ Polymer-based composite percolation thresholds can be reached relatively quickly at low concentrations (∼1 wt %) with acceptable stretchability, high carrier mobility, and good sensitivity.^112^ In flexible sensor applications, CNT is usually functionalized with elastomers such as Ecoflex, polyisoprene (PI), and PDMS.^25^ Despite the advantages, composite fabrication with CNTs faces problems of agglomeration caused by electrostatic interactions that negatively affect the performance of the device.^25^

Conductive polymers, with behavior similar to that of semiconductors, are another class of materials often used as conductive fillers with the main advantage of tunable conductivity combined with great flexibility.^25^ These composites, composed of an insulating polymer matrix functionalized with conductive polymer fillers, in which the latter provide the necessary charge carriers, are some of the most popular among sensors based on a piezoresistive principle. Intrinsically conductive polymers such as polypyrrole,^113^ PEDOT:PSS,^114^ and polyaniline (PANI)^115,116^ can be used in combination with substrate polymers such as PDMS and PET^25^ to achieve the desired compromise between flexibility and electrical properties/sensitivity. However, although these materials provide the flexibility required for sensor systems, their electrical conductivity is lower than that of the metals and carbon allotrope fillers. In any case, according to Wang et al.^117^ and Vosgueritchian et al.^118^ PEDOT:PSS is a good candidate for improving the stretchability of conductive polymer composites, upon the addition of fluorosurfactants and nonionic plasticizers.

The MXenes are a class of 2D nanomaterials with excellent electrical conductivity, unique layered structures, hydrophilicity, large specific surface areas, thicknesses in the range of 1–100 nm, and abundant terminal groups.^25,119^ MXenes are widely used in composites due to their excellent compatibility, combining or complementing the properties of the polymers with exceptional properties, especially in terms of electrical conductivity and durability, with valuable results in the detection of respiratory biomarkers, among others.^25,119,120^ However, MXenes also have some disadvantages, mainly mechanical instability and instability under oxygen, which limit the use of pure MXenes in wearable sensing devices.^24^

Metallic fillers are also quite popular in the field of wearable sensors. In this group, silver nanowires (AgNWs) appear to be the most commonly used material, which has gathered great attention due to its excellent stiffness and electrical conductivity properties, along with its potential in smart textiles, e-skins, and structural health monitoring applications.^24,121^ Other metal precursors, organometallic compounds, copper, gold, and platinum are also used due to their potential for commercial applications.^25,122^

Thus, electromechanical stability under strain, geometric designs with wavy or serpentine-shaped structures,^7^ network patterns,^37^ and 3D porous patterns and crumpled structures^38,39^ have been reported to impart high stretchability to otherwise rigid films, while minimizing strain on the conducting materials and maintaining conductivity during reversible elongation cycles.^1,29^ Another possible approach could be to mix or create hybrid materials from two or more of these nanomaterials, to provide improved electrical conductivity and stability.^122^

Nevertheless, metal fillers tend to exhibit relatively high cost, poor surface modification, oxidation tendency, nonlinearity, poor stability, low abundance, and poor acid and alkali resistance, which sometimes hinders their durability, scalability, and application in physiological signal monitoring.^24,98,121^ Thus, the development of metal fillers with optimal properties is a major research focus, while at the same time, rational electrode design and structural optimization are important requirements for improving detection efficiency and versatility in various detection environments.^24^

Finally, liquid metals are also being used because of their inherent flexibility, stretchability, and excellent conductivity and are expected to thrive when applied to prosthetic, robotic, and wearable devices that operate in particularly curved or soft surfaces.^123^ The most commonly used liquid metal is Galinstan, an alloy of gallium, indium, and tin, and eutectic gallium indium (EGaln), which is widely used in electrodes and sensors.^95,123^ Considering that both liquid metals and conductive polymers, such as PEDOT:PSS films, exhibit similar properties with the addition of fluidic behavior, various stretchable conductors with different patterns and microchannels have been designed to effectively guide liquid metals, such as PMDS and Galinstan composites. This fluidic characteristic can also lead to self-healing properties in reusable devices.^94^ Liquid metals have the ability to self-heal damage and maintain continuous electrical performance.^95^

## Flexible Sensor Manufacturing

The overall performance and quality of wearable flexible sensors can be estimated in the laboratory to detect physiological and anatomical changes in the human body. Because wearable models offer advantages over their stationary counterparts, the biomedical and bioengineering fields have recognized the great potential of these devices to capture and monitor physicochemical parameters and anomalies in humans.^121^ Typically, the process for fabricating a patterned substrate or matrix is simple, where a substrate such as PDMS is poured into a previously fabricated micropatterned mold, followed by curing and peel-off processes.^25^

However, with other materials, structures, and dimensions, the manufacture of the sensors can vary greatly.^121^ In the fabrication of flexible sensors, especially with regard to composite materials, the substrate/matrix acts as a flexible support, providing the required stretchability and mechanical flexibility, while active layers or conductive fillers are responsible for the transduction mechanisms and complement the properties of the matrix. Thus, researchers have developed various manufacturing processes that can improve nanomaterial functionalization and overall sensor performance, including the use of more efficient uniform mixing methods, along with ordered structures, such as nanofibers, films, yarns, fabrics, and foams, among others, with the sensor properties shifting depending on the manufacturing approach.^124^

2D active film sensing systems consist of a substrate patterned with 0D or 1D dimensional nanomaterials in a single or multilayer periodic structure. In the case of multiple layers, the same process can be performed two or more times on the same substrate, but in different directions. The functionalization methods used in the preparation of active films can be classified into coating, electrospinning, assembly, transfer printing, and oriented growth approaches.^125^

### Coating

**Figure 8:**
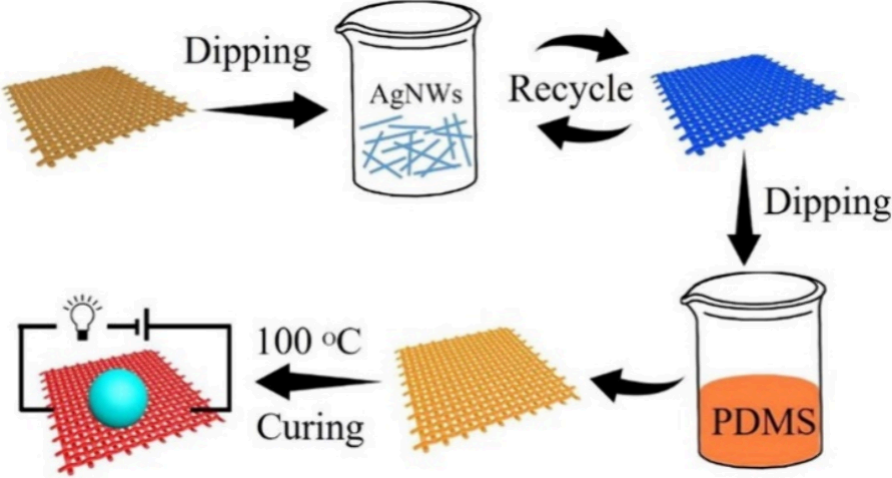
Schematic illustration of a AgNW@PDMS composite fabrication via a dipping–thermal curing method. Reprinted with permission from ref (128). Copyright 2019, Elsevier B.V.

Coating methods are widely used because of their simplicity in producing active films on a large scale. In this approach, nanomaterials such as nanowires, nanotubes, and nanorods are dispersed in coating solutions and deposited onto the substrate. By applying a shear force, the nanomaterials, which would otherwise have random orientations, can assume the desired orientations.^126^ One of these coating methods is direct coating, in which a dispersion of 1D nanomaterials is deposited drop by drop onto a substrate. During this process, a brush, Mayer bar, or other similar tool is dragged through the dispersion at a constant speed, orienting the nanomaterials parallel to the direction of the drag due to the resulting shear forces generated by these tools.^125^ Direct coating is a straightforward method for fabricating 2D micro/nanostructures because the orientation is directly influenced by the viscosity of the solvent, the aspect ratio of the chosen nanomaterials, and the drag speed. Nevertheless, limitations in coating tool technology can limit the overall process, as the regularity of the resulting structures is very poor, with the need for postprocess realignment of the nanomaterials being common.^127^ Another method is dip-coating, a process in which the desired substrate is dipped into a dispersion of the conductive nanomaterial and then aligned, while the resulting structure is lifted out of the dispersion by gravity, as shown in Figure 8. In this method, the alignment of the conductive nanomaterials can be facilitated by the flowing solution, depending on the lifting rate of the substrate, the viscosity of the solution, and the evaporation rate. Considering the wide range of applications and potential candidate substrates, together with the low consumption of conductive nanomaterials, dip-coating has attracted a lot of attention as an effective approach to fabricate films based on 1D nanomaterials. However, one issue that may arise is the lack of alignment control because the conductive nanomaterials are deposited on the substrate surface in a random manner.^128^

### Electrospinning

Electrospinning is a promising method that focuses primarily on the production of ultrahigh aspect ratio nanofibers. When a strong electric field is applied between the metal needle and the collector, the solution containing the desired nanomaterial is pulled toward the collector via electrostatic forces, creating nanofibers. Unfortunately, due to the instability inherent in the process, the nanofibers produced are not aligned and have a completely random arrangement.^129^ Several approaches have been attempted to align nanofibers produced by electrospinning, including modifications to the collector or the application of a magnetic field.^130−133^

Regarding collectors, two types have been used to prepare nanofiber-based active films. One is to rotate the collector at high speeds by using a drum-shaped collector, which induces unidirectional alignment of the nanofibers due to the high shear forces generated.^132,134^ However, the rotation speed must be carefully calculated: low rotation speeds result in poorly aligned nanofiber yields and high rotation speeds can break the nanofibers.^125^ Other method, according to Maity et al.,^135^ is to add two parallel electrodes to the collector, which creates two parallel electric fields that straighten the nanofibers so that they have an orientation angle of between 60° and 90° relative to the electrodes.

However, some of the challenges of this approach include limitations on the materials that can be electrospun, because polymers such as PP, PE, and PA require mixing with solvents at high temperatures.^81^ Since most solvents are hazardous to workers and may be present in the final product, applications may be limited.^81^ In addition, the electrospinning of certain nanomaterials, such as inorganic nanofibers, results in a high degree of brittleness, which leads to short lifetimes even though the sensors have high sensitivity and short response times.^81^ Another problem is the time required for this process, which severely limits the potential of this technology for large-scale production of sensor batches. This is an issue that needs addressed as a matter of urgency.^81^

### Assembly Methods

With respect to the assembly functionalization methods, this induces arrangements and aggregations of previously randomly oriented nanomaterials by applying external forces, such as electric fields,^136^ tensile stress,^137^ and shear forces,^138^ among others, or by creating attractive^139^ and repulsive^140^ interactions between the conductive nanomaterials, resulting in a yield of well-aligned films with a regular pattern. In contrast to the coating methods, which produce films of conductive nanomaterials that are poorly aligned and lacking in regularity, in this method, the orientation can be directly controlled by external forces, with significantly higher regularity.^137^ In addition, the application of electromagnetic fields has the ability to assemble conductive or semiconductive nanowires, generally made of gold, silver, silicon, or zinc oxide, and other nanomaterial dimensionalities into aligned and organized structures.^125^

A challenge for sensors produced by these processes is to understand all the factors that influence the final stretchability and durability in on-skin applications.^141^ This requires control of the process at the molecular level to effectively align the nanomaterials, which is difficult in some techniques.^125^ Another issue is the complex relationship between the applied strain in the nanostructure and its electromechanical properties, where multiple factors may negatively affect performance, including the chemical nature of some materials, such as metals and nanoparticles, oxidations,^141^ chosen geometries, particle size, stability under prolonged use, adhesion issues, mechanical cracks, and moderation of the process when using thermally or mechanically sensitive substrates, including polymers and glasses.^141,142^ The study of the interactions and possible synergies between nanomaterials in nanocomposites calls for more optimized flexible electronics configurations.^141^

Finally, by focusing on self-assembly methods to benefit from greater versatility, research could also focus on exploring materials other than polymers, such as metals, ceramics, and glasses, which are still relatively rare in current work.^142^ Understanding all these mechanisms could lead to a wider range of wearable sensors and applications in various fields.

### Transfer Printing

**Figure 9:**
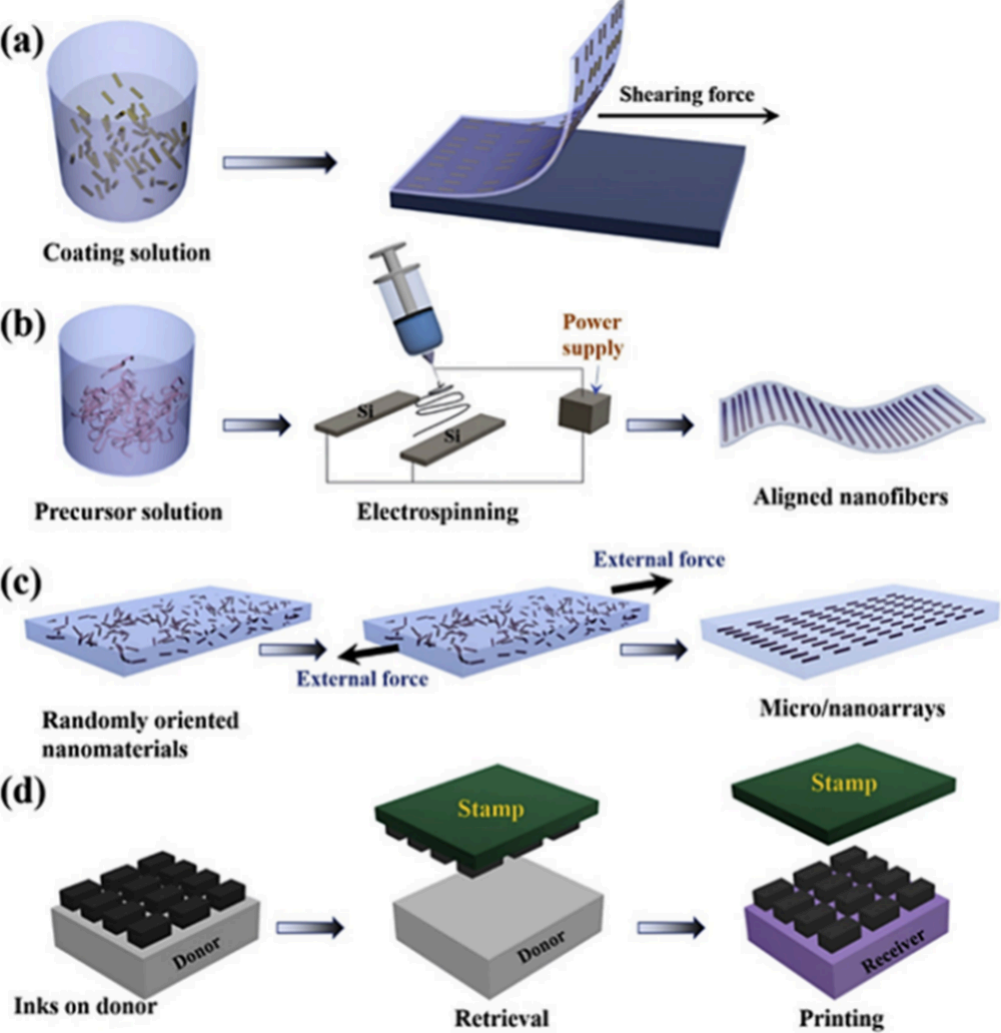
Preparation of 2D active sensing films, by functionalization through various methods: (a) coating; (b) electrospinning; (c) assembling; (d) transfer printing. Reprinted with permission from ref (125). Copyright 2022, Elsevier B.V.

Transfer printing is another approach in which previously synthesized conductive nanomaterials, typically with random orientations, are detached from a donor substrate, reorganized, and deposited onto a receiver substrate, resulting in aligned nanomaterial patterns. The transfer method can work by directly contacting the receiver and donor substrates, or by using a stamp as an intermediary. According to Lin et al.,^125^ ideally, any transfer printing process should meet two key requirements: achieving the desired alignment of the nanomaterials on the surface of the substrate and ensuring that nanomaterials readily release from the donor and adhere to the receiver. For the first requirement, a manufacturing design that uses external forces, such as friction or capillary forces,^143^ as a support mechanism can improve the overall alignment of the nanomaterials. For the second requirement, the entire process depends on eq 3(125)
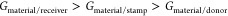
3where *G* is the adhesion strength of the nanomaterial to each one of the elements of the process. Only by respecting this expression can a defect-free transfer of the nanomaterials to the recipient substrate be achieved. Although finding materials that meet this requirement is a difficult task, some physical strategies can facilitate the entire process, namely the use of leaf-shaped stamps and gecko-inspired structures. Due to the increased contact area between the nanomaterial and the leaf, there is a higher adhesion compared to regular stamps, which press the nanomaterials perpendicularly. Moreover, the nanomaterials have a tendency to detach from the leaf-like structure when it is retracted.^125^Figure 9 illustrates the functionalization methods that enable the fabrication of active-sensing 2D films.

Regarding the 3D sensor structures, they can be fabricated from 3D micro/nanomaterials, such as domes, spheres, pyramids, walls, rods, pillars, sheets, and others. In addition, hierarchical structures can be obtained by surface modification of the nanomaterials. The main methods used to fabricate 3D sensing structures are epitaxial growth, template methods, carved templates, and bionic approaches.^125^

### Screen Printing

Screen printing is a promising technique in the wearable sensor field and is one of the most popular printing approaches, allowing for a simple and versatile production of the desired patterns at a reduced cost in multiple substrates.^144^ This is due to a high degree of functional layer compatibility, pattern design flexibility, environmentally friendly processes, and large-scale potential, providing a useful functionality with multiple types of conductive inks, such as silver and carbon pastes, dielectric inks, and a large myriad of functional materials.^145,146^

In this technique, the conductive ink is first poured in the upper surface of a woven screen made of synthetic fibers or steel mesh and then spread throughout the screen and repeatedly pressed into the substrate with a squeegee. After this step, the screen is removed and the resulting pattern, along with the substrate, must be heated to ensure that the solvents evaporate and the coating cures properly.^145^

However, there are several challenges associated with this approach, including consistently maintaining high-viscosity and low-volatility inks, that prevent self-flow through the mesh due to gravity, developing high-resolution printing techniques that increase sensor performance while addressing miniaturization trends, formulating screen-printable composites for human detection and monitoring applications, and controlling other parameters, such as screen-to-substrate distance, mesh size, and the squeegee scanning speed and pressure.^144,145^

### Epitaxial Growth

Nanomaterial synthesis can be achieved by hydrothermal methods, where the substrate is immersed in a solution of the nanomaterial, until seeds are formed on it. This is followed by continuous *in situ* growth and calcination or annealing treatments, leading to the formation of 3D nanomaterial structures.^147^ Considering this approach, *in situ* growth is mainly influenced by the time and temperature of reaction, together with the nanomaterial of choice, parameters that control the general morphology of the structure.^125^ To obtain hierarchical structures, epitaxial growth can be executed twice. Epitaxial growth is a relatively simple method for synthesizing 3D active structures due to the small number of steps required, making it a suitable candidate for large-scale production and applications. However, long reaction times and large amounts of reactants are required to achieve the desired growth and produce highly uniform structures.^125^

Most of the challenges revolve around process control and substrate selection, as both affect the quality of nanomaterial deposition and some substrates are not suitable for growth.^148^ The majority of methods used are physical, with a significant lack of chemical methods such as chemical solution deposition (CSD) and chemical vapor deposition (CVD).^148^ Controlling the surface roughness of the film is another challenge, requiring optimization of the substrate/reinforcement interface. Maintaining adhesion between the substrate and the reinforcement can be an issue that can hinder the effectiveness of some techniques, depending on the flexibility of the materials chosen.^148^

### Template-Based Methods

The support provided by a template allows the deposition and growth of nanomaterials in the same direction, forming the desired patterns, followed by the removal of the template when the process is complete.^149^ Anodic aluminum oxide (AAO) is the most commonly used template, especially for the preparation of pillar-shaped structures. This is the case due to the postmodification-friendly pore size of the templates, typically between 10 and 400 nm, along with rigid pores that are densely packed, allowing for the formation of well-defined nanostructures. The high thermal stability of AAO allows physicochemical processes to occur at high temperatures, while the hydroxylated pore walls of AAO allow adequate infiltration of polymer solutions.^150^ When a polymer solution is poured onto an AAO template, the solution rapidly permeates, resulting in solid polymer nanopillars after the solvent evaporates. Thus, this approach has exceptional potential for self-powered sensor applications.^125^ Templates with complex nanostructures, including nanopillars and pyramids, can be fabricated using carving technologies such as photolithography. Nonetheless, due to the inherent high cost and complex nature of this process, simpler methods are being explored, including drilling holes in hard substrates.^151,152^

Despite this, some template methods have higher structure regularity than others, such as AAO templates, some have relatively difficult fabrication procedures, such as carved templates, while others are not suitable for large-scale production and excel more at the prototyping level, which includes self-assembling templates and carved templates.^125^ Additionally, when using spherical nanoparticles, due to their isotropic nature, they could aggregate randomly in nonsolvents, requiring specific template methods to correct this behavior, such as ice templates.^125^ These are some of the factors that should be considered when choosing the manufacturing approach to scale-up technology.

### Bionic Templates

Structures such as as leaves and petals are used, which are optimal candidates as natural templates due to their versatile and aligned nanostructures. Some examples are the surface of lotus leaves,^153^ which have a large number of micropapilla-like structures on their surface, and *Acacia mill* leaves, which have needle-like microstructures with a high aspect ratio since the diameter is ∼25 mm and the length is ∼300 mm. According to Wan et al.,^154^ after lotus leaves are dried their internal structure and surface morphology can be retained to be used as templates for the fabrication of dielectric layers for sensors.

For strain and pressure sensing common methods for achieving flexibility and stretchability with adequate sensing performance involve the dispersion of conductive fillers within an elastomeric matrix. These include shear mixing, *in situ* polymerization, and solution mixing, namely, the surface functionalization and the addition of surfactants to achieve more uniform dispersions. Depending on the morphology and shape of the composites, they can be further processed by spinning, compression molding, extrusion, and film casting. It should be noted that the transduction mechanisms of these composites can be adjusted by varying the concentration of conductive filler in the matrix to obtain a stable compromise between mechanical stretchability and electrical conductivity.^98^

As for the limitations, in addition to the environmental impact of some methods, there are still complications in controlling the uniformity of the film and issues related to the bonding interaction between the conductive filler and the matrix, which could lead to the delamination of the conductive layer.^98^

In summary, although the correct choice of matrix and conductive filler is essential to achieve optimum performance, it is the functionalization and fabrication methods that have the greatest impact on the final performance of the sensor. For these reasons, the search continues for cost-effective, mass-producible, and practical methods that maintain a degree of simplicity and are not time-consuming.^98^

## Case Studies and Trends

This section aims to identify research trends based on the analysis of works from the last 5 years, chosen materials, preferred operational methodologies, and fabrication and functionalization techniques, for flexible sensors of temperature, humidity, strain/pressure, and sweat. In addition, the resulting performance of each sensor is summarized, and the defined parameters are correlated with the results obtained.

### Temperature Sensors

**Figure 10:**
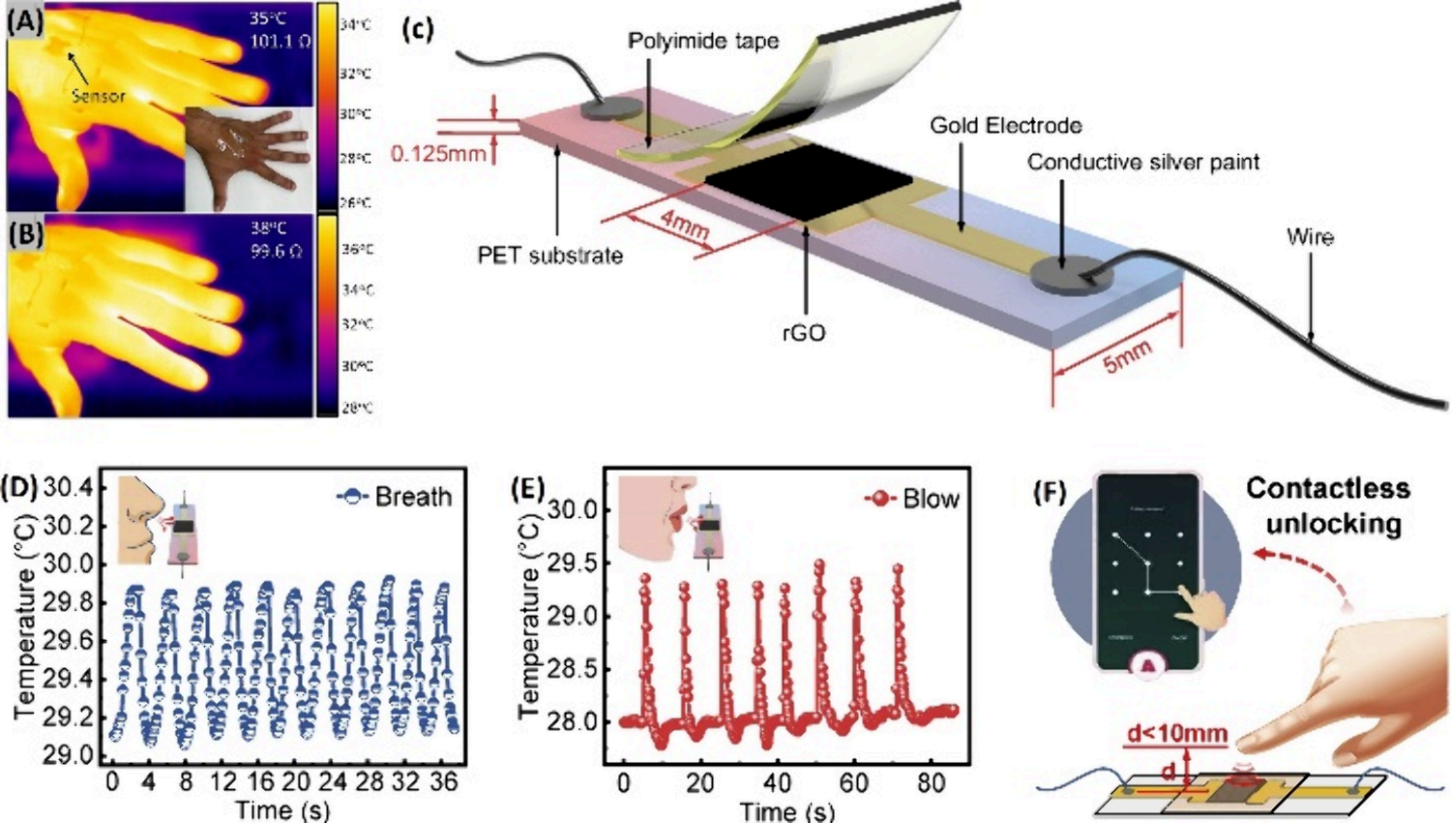
(A, B) Thermal images of the back of the hand acquired via an IR camera at temperatures of 35 and 38 °C. The position of the sensor is indicated by the arrow, and the sensor attached to the hand is shown in the inset. Reprinted with permission from ref (155). Copyright 2021, Elsevier B.V.. (C) Schematic of the temperature sensor. (D, E) Sensor application test for breathing rate monitoring and human blowing detection. (F) Proximity detection experiment. Reprinted with permission from ref (156). Copyright 2022, Elsevier B.V.

Ben-Shimon et al.^155^ developed a flexible and biocompatible temperature sensor based on a PDMS@CNT composite for monitoring body temperature. The potential of these on-skin applications was demonstrated by using a CNT forest design to sense thermal variations between the matrix and the reinforcement, inducing changes in electrical resistance. The chosen operating principle leads to a simpler fabrication process and sensing scheme, along with excellent performance, biocompatibility, and stability and reproducibility during long-term operation. The device also showed high resilience to both mechanical and sequential thermal stress, with sensitivity up to 0.01 Ω/°C and a stable Young’s modulus of ∼0.1 MPa at 100 °C. The sensitivity is higher than the average of the sensors reported in the literature (∼1 Ω/°C), with electrical resistances of 101.1 and 99.6 Ω at 35 and 38 °C, respectively (Figure 10A,B).

In the work of Chen et al.,^156^ a fast-responding flexible temperature sensor based on laser-reduced GO was fabricated by drop-coating for a noncontact human–machine interface (Figure 10C). The influence of both processes, along with the GO concentration, on the final sensitivity and response times was investigated. The fabricated sensors exhibited good linearity, stability, low hysteresis, good repeatability, and response times of 0.196 and 0.274 s for sudden temperature increases and decreases, respectively, shorter than those of most rGO-based temperature sensors. In addition, the sensor was able to monitor human breathing, detect blowing and human fingertips, and surface contactless unlocking of a mobile phone (Figure 10D–F). The results showed that a GO concentration of 4 mg/mL had the highest sensitivity, of 0.37%/°C, between 30 and 100 °C, and superior linearity, *R*^2^ = 0.996. The sensor had a good temperature response, and the recovery time was ∼9.7 s at the heating–cooling cycle in the 30–50 °C range.

Zhu et al.^157^ developed a flexible temperature sensor based on a TPU@SWCNT composite via solution mixing and thermal annealing for respiration monitoring, differentiating between deep and normal breathing, and noncontact temperature sensing applications. The sensor exhibited a relatively linear NTC effect in the temperature range of 30–100 °C, with excellent reproducibility, high reliability, and an accuracy of ±0.1 °C, without affecting the thermal response by deformation and heating rate, after at least 200 bending cycles. During composite fabrication, electrical conductivity was observed to increase and stabilize after 2 h of thermal annealing. The relative resistance variation decreases linearly with temperature increments, exhibiting an NTC effect, with SWCNT contents of 0.25 and 0.5 wt % showing greater dependence of relative resistance variation with temperature and greater sensitivity than the other contents. Lastly, the sensor also showed electrical resistance to infrared radiation due to the excellent thermal effect of SWCNT filler.

Wang et al.^158^ reported a wearable and flexible temperature sensor developed by integrating polybutylene terephthalate (PBT) with rGO and CNT, via ultrasonication, hydrothermal reduction, and dip-coating, for human body and environmental temperature monitoring applications. The produced sensor was able to detect body temperature when placed on the forehead, palm, and back of the hand, as well as temperature variations caused by blown air, thus consolidating its potential in the field of human medicine and daily temperature monitoring. Using these methods, both rGO and CNT were uniformly loaded on the PBT surface, forming a continuous and stable conductive network with high sensitivity (−0.737%/°C), linearity (*R*^2^ = 0.98), and accuracy (0.1 °C), between 25 and 45 °C. Moreover, a response time of 31 s, stability within 300 s, and good repeatability were observed while monitoring human body temperature around 37 and 38 °C and respiration rate and detecting room temperature in the range of 25–45 °C. Compared to pristine PBT the mechanical properties of the sensor were improved, along with long-term stability and a signal with good circularity.

Geng et al.^159^ produced a wearable and tunable temperature sensor by solution blending of acrylate copolymers with CB for smart, wearable, and adjustable temperature sensor applications. The system presented high sensitivity, fast response times, an accuracy of 0.5 °C, a relative resistance variation of 12.5% per 0.5 °C, and stability over 200 heating cycles, with the cold and hot cycle lasting 20 s. The device was highly sensitive to temperature variations in a wide range of values; the electrical resistance changed by nearly 3 orders of magnitude when the temperature changed from 33 to 40 °C by and 4 orders of magnitude when the temperature was increased from 25 to 40 °C. Since these characteristics enable real-time temperature monitoring, an LED bulb was integrated into the device to turn on and off as the temperature increased or decreased. Lin et al.^160^ proposed a flexible thermocouple temperature sensor based on an alumina–silicon oxide aerogel as a substrate and indium oxide (In_2_O_3_) and indium tin oxide (ITO) as active materials by a screen-printing technique for aerospace, metallurgy, and explosion damage detection applications, among others. The fabricated sensor operated under harsh conditions over a very wide temperature range from −196 °C up to 1200 °C, with a sensitivity of up to 226.7 μV °C^–1^, a maximum peak-to-peak output voltage of 0.23 mV, a repeatability error of ±1.72%, and a response time of ∼5 ms, meeting the requirements of daily life, as well as laser processing and construction machinery, with characteristics that are difficult to achieve by other sensors in this field.

Geng et al.^159^ synthesized a body temperature sensor based on a polyimide film with drop-cast carbon black and acrylate copolymer, all encapsulated in PDMS, tunable for a temperature range of 33–40 °C, indicated for body temperature monitoring and other smart wearable electronics applications. The produced sensors exhibited an electrical resistance change of more than 3 orders of magnitude from 30 to 40 °C and 4 orders of magnitude between 25 and 45 °C, at a ramp rate of 10 °C/min. In addition to no observable NTC phenomenon, the sensors maintained a sensitivity of 3 orders of magnitude after 200 heating cycles, showing higher sensitivity when the sensor was thicker and faster recovery time when the sensor was thinner. The temperature response performance was highly repeatable, fast, and stable, while responding regularly and quickly to temperature changes and maintaining stable resistance up to 1600 s. The temperature coefficient resistance (TCR) was up to 6.2% °C^–1^, which means that the temperature can be monitored within a 1 °C change. Lastly, temperature changes can be visualized by turning an LED bulb on above 33 °C and off above 40 °C.

Sun et al.^161^ fabricated a flexible hydrogel-based temperature and strain sensor by solvent casting, with a gelatin and polyacrylamide substrate with silver nanowires, for strain/pressure, body temperature, and human motion monitoring applications. The produced sensors showed a strain capacity and stretchability 2.6 and 1.9 times higher that of than pure gelatin, respectively, with the Young’s modulus increasing from 111 to 171 kPa. The hysteresis increases with higher tensile strain. In terms of electrical properties, the conductivity of the sensor reached a maximum of 0.0056 S/m, with a slight drift in the signal after 400 cycles at 5% strain. The gauge factors obtained were 0.7 at 150% strain and 1.8 at 658% strain. The working range was 1–658%, with a detection threshold within 1% strain. Response and recovery times were 187 and 703 ms, respectively. The TCR values were −8.0 °C/% at 6–15 °C and −0.7 °C/% at 15–36 °C.

From the summarized information in Table 2, it can be observed that composites made up of different polymeric matrices (PDMS, PET, TPU, PBT, and acrylate) and conductive fillers (CNT, SWCNT, rGO, and CB) resulted in flexible sensors with good response, especially high sensitivity and linear behavior at temperatures below 100 °C.

**Table 2:** Featured Published Works for Flexible Temperature Sensors

key materials	application	main properties	ref
PDMS@CNT	body temperature monitoring	CVD + molding; sensitivity up to 0.01 Ω/°C; Young’s modulus of ∼0.1 MPa at 100 °C	(155)
PET@rGO	contactless human–machine interface	laser-reduction + drop-coating; response and recovery times of 0.196 and ∼9.7 s; sensitivity of 0.37%/°C	(156)
TPU@SWCNT	respiration monitoring and noncontact temperature detection	solution blending + thermal annealing; linear NTC effect between 30 and 100 °C; high reliability and an accuracy of ±0.1 °C	(157)
PBT@rGO/CNT	human body and environment temperature monitoring	ultrasonication + hydrothermal reduction + dip-coating; high sensitivity of −0.737%/°C; linearity of *R*^2^ = 0.98; accuracy of 0.1 °C	(158)
acrylate copolymer@CB	smart wearable and adjustable temperature sensors	radical polymerization + solution melting; Accuracy of 0.5 °C;	(159)
		Resistance variation of 12.5% per 0.5 °C.	
alumina-silicon oxide@In_2_O_3_/ITO	body temperature monitoring, aerospace, laser processing	screen-printing; sensitivity up to 226.7 μV °C^–1^; range from −196 °C up to 1200 °C; repeatability error of ±1.72%; peak-to-peak output voltage of 0.23 mV; response time of ∼5 ms	(160)
PI+PDMS@CB+acrylate copolymer	body temperature monitoring and other smart wearables	drop-casting; temperature range between 33 and 40 °C; sensitivity up to 3 orders of magnitude from 30 to 40 °C; stability up to 200 heating cycles; TCR up to 6.2% °C^–1^; temperature detection within 1 °C	(159)
gelatin+polyacrylamide@AgNW	body temperature and motion monitoring	solvent-casting; Young’s modulus up to 171 kPa; conductivity of 0.0056 S/m; gauge factor of 1.8 at 658% strain; response and recovery times of 187 and 703 ms; TCR of −0.7 °C/% at 15–36 °C and −8.0 °C/% at 6–15 °C	(161)

### Humidity Sensors

**Figure 11:**
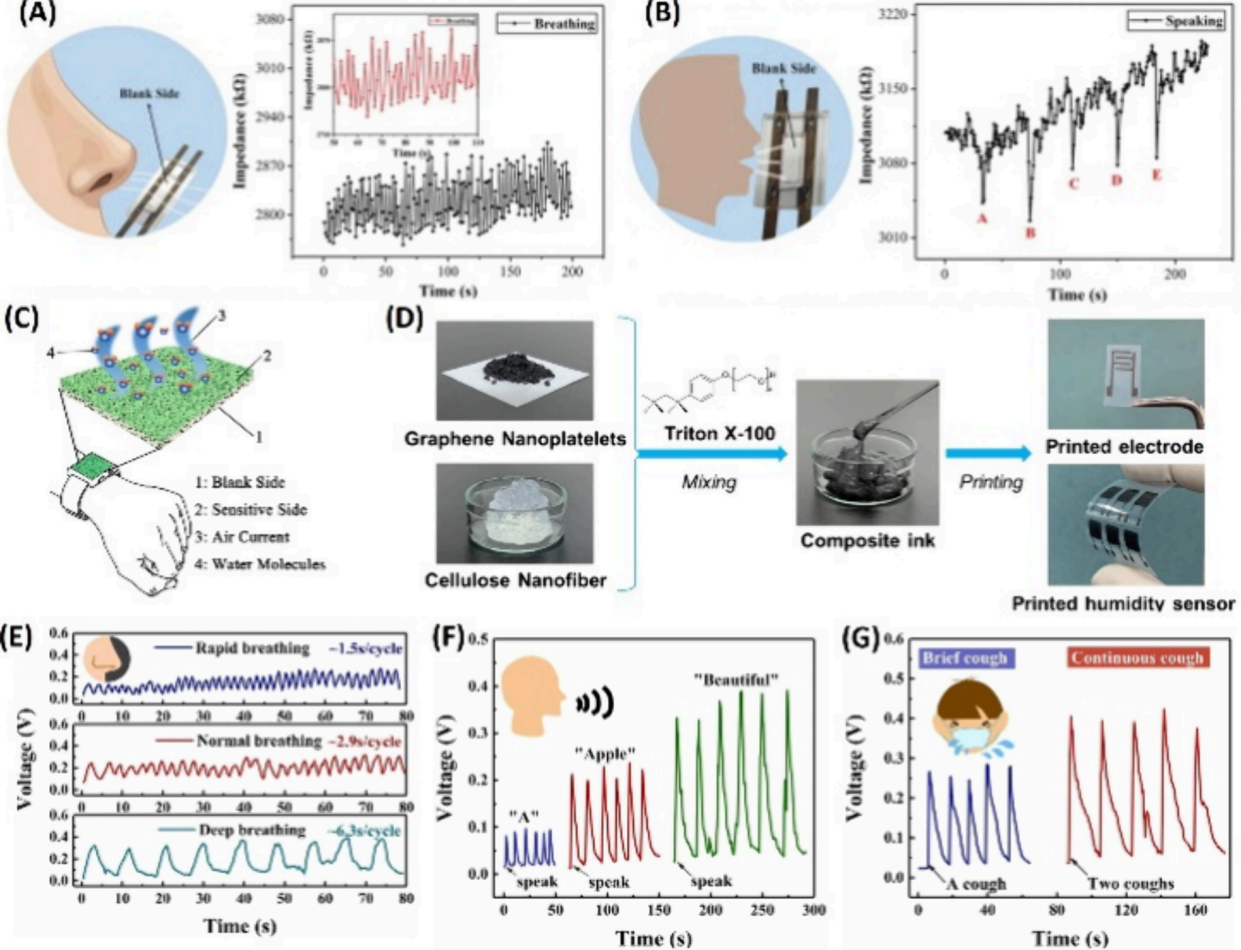
(A, B) Impedance response PANI/PVDF for breathing and speaking. (C) Schematic illustration of the working principle of the PANI/PVDF-based device. Reprinted with permission from ref (162). Copyright 2022, Elsevier B.V. (D) Preparation of CNF/GNP composite inks and images of printed electrode and printed sensors on PEN film. Reprinted with permission from ref (163) under a Creative Commons Attribution License 4.0 (CC-BY) (https://creativecommons.org/licenses/by/4.0/). Copyright 2022 The Authors. Published by Elsevier B.V. (E–G) Voltage response of the sensor for various nose respiration rates, various pronunciation syllables, and brief and continuous coughing. Reprinted with permission from ref (164). Copyright 2023, Elsevier B.V.

Zhao et al.^162^ developed a single-sided, flexible, nontoxic, and breathable humidity sensor based on a PVDF@PANI composite, for breathing and speaking monitoring applications (Figure 11A,B). PANI was unilaterally deposited on a microporous PVDF matrix by *in situ* polymerization and presented good humidity sensing properties at room temperature, including small hysteresis (∼5% RH), a detection range of 11–98% RH, and a relative resistance variation up to 226%, along with a reversible, fast, and stable response, even under bending deformation. The unilateral deposition of PANI minimizes the contact between the material and human skin, preserving the humidity-sensing characteristic of the former while avoiding damage to the latter (Figure 11C). Additionally, the functionalization method avoids the need to further integrate a patterned interdigital metal electrode into the structure. The prepared 0.01 mol/L PANI/PVDF humidity sensor showed hysteresis and response times comparable or superior to those of other reported flexible sensors, and stable responses during deformation, a characteristic rarely found in flexible humidity sensors.

In Yoshida et al.,^163^ a printed flexible humidity sensor based on a dry-blended cellulose nanofiber/graphene nanoplatelet (GNP) composite ink, screen-printed on a PEN substrate, was produced for human respiration and skin moisture monitoring applications. This sensor exhibited a high resistance response of 240% over the relative humidity range of 30–90% RH, with response and recovery times of 17 and 22 s, respectively, along with good mechanical flexibility. Due to the abundance of cellulose and GNP in nature, this work aimed to produce a cost-effective, environmentally friendly, degradable, and biocompatible humidity sensor with high performance. Regarding the fabrication of the composite ink, GNP was easily dispersed into the cellulose nanofibers using a planetary mixer, without sonication or other complex surface modifications to maintain the ability of the composite to be screen printed. In addition, the need for electrode integration was eliminated because the composite ink was used as both the sensing layer and the electrodes, eliminating the need for gold and silver (Figure 11D). Due to these factors, coupled with a relative resistance variation of 2.4 along the 30–90% RH range, the authors concluded that the produced sensor is a promising candidate for the new generation of IoT technologies.

Guo et al.^164^ presented a self-powered flexible humidity sensor based on a unique sandwich structure of PVA/nanocarbon powder (NCP)/MgCl_2_ fabricated by solution casting for human respiration monitoring applications. The sandwich structure allowed this sensor to readily adsorb and diffuse water molecules from the environment, achieving a response linearity of *R*^2^ = 0.9978, response and recovery times of 6 and 11 s, respectively, a sensing range of 11–98% RH, stability for over 30 days, along with high voltage and current outputs of ∼0.6 V and ∼2.3 μA, and a calculated power of ∼1.38 μW, at 98% RH. Additionally, due to the good water solubility properties of PVA and MgCl_2_, the sensor can be recycled and reused with up to 90.31% of the original response voltage value, dramatically minimizing material waste and reducing overall manufacturing costs. The sensor has also been integrated into a mask to monitor human breathing, distinguishing between different breathing rates and patterns, as well as detecting human speech and coughing (Figure 11E–G).

In the work of Liang et al.,^165^ a flexible humidity sensor based on a prestretched PDMS substrate integrated with rGO was developed for breathing pattern and respiration monitoring applications. The sensor was produced with rGO in a wrinkled structure, which improved both response and recovery times, while preventing water aggregation and condensation, and shortening water adsorption and desorption times. This structure provided flexibility while maintaining the characteristics of the sensor during deformation. This made the sensor suitable for on-skin monitoring applications. In terms of results, it was concluded that the thickness of the rGO correlated with response and recovery times, which could be further accelerated by reducing the thickness of the rGO. Thicker rGO exhibited response and recovery times of 6 and 7.5 s, respectively, while the thinner rGO had times of 2.4 and 1.7 s, respectively. Stability and repeatability for more than 30 cycles were achieved, along with high sensitivity in the 11–95% RH range. Maximum wet hysteresis was observed at 85% RH, with the maximum value of 3% RH, demonstrating the capabilities of wrinkled structures.

Li et al.^166^ fabricated a capacitive humidity sensor based on an indium oxide (In_2_O_3_) and GO film integrated on an epoxy substrate, which was developed for monitoring breathing patterns and respiratory diseases, including asthma and other complications. This system exhibited portability, accuracy, immediacy, and low power consumption, along with other characteristics that have been improved compared to pure In_2_O_3_ or GO, including high stability, repeatability, lower response and recovery times, and higher sensitivity. In terms of results, humidity sensing capabilities were examined over a range of 11–97% RH at 20 °C. As RH increased, a significant increase in the capacitance response of the sensor was observed in real time, with particularly high sensitivity and capacitance changes at lower RH values, between 11 and 23%. The response and recovery times of the composite film were shorter than those of the single materials, at 15 and 2.5 s, respectively. The maximum hysteresis value was 0.054% RH, observed at 85% RH.

Cui et al.^167^ developed a wearable capacitive humidity sensor based on a polyimide substrate + liquid-metal gallium(III) oxide system (Ga_2_O_3_/LM), manufactured via a laser direct writing technique, for respiration rate and skin moisture monitoring applications. The sensor exhibited a resistivity of 0.19 Ω cm, at a laser fluence above 6.8 J/cm^2^ and a maximum capacitance change of 136.3% in the 30–95% RH range, with an electrode width of 1.5 mm and a temperature of 20 °C, as well as a highly stable cycling stability, where no significant degradation was observed at the end of 46 h of continuous use. When used to monitor respiration patterns, a capacitance change of 193.1% was recorded over five measurement periods, along with a response time of ∼1.2 s and a recovery time of ∼1.6 s.

Guo et al.^164^ reported a self-powered flexible humidity sensor based on a sandwich-like structure consisting of PVA, nanocarbon powder, and magnesium chloride (MgCl_2_), manufactured via solution casting, for human respiration and speech monitoring, along with noncontact switching devices, among other wearable sensor applications. The manufactured sensors displayed excellent linearity (*R*^2^ = 0.99781) and sensitivity (∼9.2 mV/% RH), along with a working range of 11–98% RH. The output voltages at 11% RH and 98% RH were 0.7 and 0.6 V, respectively, with an output current of ∼2.3 μA and power of ∼1.38 μW, showing that the sensor responds well to changes in ambient humidity. The observed response and recovery times were 6 and 11 s, respectively, and according to the authors exhibited better sensitivity, operating range, output voltage, and response characteristics than many sensors in the field. Regarding durability and stability, the output voltage is stable in both flat and bending states, while maintaining a stable cyclic voltage response for up to 10000 s in a 98% RH environment. Furthermore, the sensors could be successfully recycled and reused, with the devices retaining 90.31% of their original response voltage and 83.51% of their response current after a dissolution and remodeling process. The sensor was able to detect pronounced words, coughing, skin moisture, approaching fingers, and oral and nasal breathing patterns.

Zhao et al.^168^ prepared a flexible capacitive humidity sensor based on a “pine nut” microstructure composed of two intertwined copper wires coated with soluble PI and poly(glycidyl methacrylate) (PGMA), for real-time monitoring of water content in liquids and air applications. The sensor exhibited a sensitivity of ∼1.4%/% RH and a hysteresis of 6.6% RH, with sensors prepared with higher concentrations of PI revealing the minimum hysteresis values. The working range of the tests was 11–98% RH, where the sensor displayed a fast response to changing ambient humidity, with a response time of 10.1 s and a recovery time of 5.2 s, along with negligible capacitance changes when bending is applied (≲0.5%). The device showed stable capacitance for up to 30 days of testing at ambient temperature, with good enough performance to detect humidity in the air. It also detected 122, 279, 1357, and 87 ppm of water in transformer oil, hydraulic oil, ethanol, and *n*-dodecane, respectively.

Table 3 shows a summary of the flexible humidity sensors reported. From the analysis of these works, a trend can be observed, including detection ranges over wide relative humidity ranges and low response and recovery times, as well as high values of relative electrical resistance variation.

**Table 3:** Featured Published Works for Flexible Humidity Sensors

key materials	application	main properties	ref
PVDF@PANI	breathing and speech monitoring	*in situ* polymerization; detection range of 11–98% RH; relative resistance variation up to 226%	(162)
PEN@cellulose nanofiber/GNP	human respiration and skin moisture monitoring	dry blending+screen-printing; detection range of 30–90% RH; relative resistance variation of 240%	(163)
PVA@CNP/MgCl_2_	human respiration monitoring	solution casting; detection range of 11–98% RH; response linearity of *R*^2^ = 0.9978	(164)
PDMS@rGO	breathing patterns and respiratory monitoring	Hummer’s method; high sensitivity in the 11–95% RH range; response and recovery times of 2.4 and 1.7 s	(165)
epoxy@indium oxide (In_2_O_3_) /GO	breathing patterns and respiratory diseases monitoring	hydrothermal method; response and recovery time of 15 and 2.5 s; maximum hysteresis value of 0.054% RH, at 85% RH	(166)
PI@Ga_2_O_3_	respiration rate and skin moisture monitoring	laser direct writing; maximum capacitance change of 193.1%; working range between 30 and 95% RH; resistivity of 0.19 Ω cm; response and recovery times of ∼1.2 and ∼1.6 s	(167)
PVA@nanocarbon powder+MgCl_2_	breathing and speech monitoring; noncontact switches	solution casting; sensitivity of ∼9.2 mV/% RH; Llinearity of *R*^2^ = 0.99781; maximum output voltage of 0.6 V; response and recovery times of 6 and 11 s; working range of 11–98% RH	(164)
Cu@PI+PGMA	real-time monitoring of water content in the air and liquids	sensitivity of ∼1.4%/% RH; hysteresis of 6.6% RH; working range of 11–98% RH; response and recovery times of 10.1 and 5.2 s; stability up to 30 days of testing	(168)

### Strain/Pressure Sensors

With regard to pressure sensing, Du et al.^169^ proposed a systematic study of piezoresistivity, electromechanical behavior, impedance, conductivity, morphology, and Young’s modulus of PDMS@MWCNT composites with crescent-shaped MWCNT contents (1–10 wt %), prepared by dry blending followed by mold casting, for monitoring human motion, such as finger, foot, and arm movement, at high strain, up to 40%. The results showed that the piezoresistive sensitivity varied inversely with the increase of the conductive reinforcement of the conductive reinforcement, and the sample with 8 wt % MWCNT showed the best sensitivity and linearity at 40% strain. By using the dry blending method, the percolation threshold was reached at ∼2 wt %, so the 3 wt % PDMS@MWCNT composite showed the highest sensitivity to strain but also showed a narrowed linear range (15–25%) and high amounts of background noise, limiting its application. As the MWCNT content was increased, both the linear range and mechanical properties were dramatically improved. Thus, it was concluded that the 8 wt % PDMS@MWCNT sample had a piezoresistive linear range between 0 and 40%, while exhibiting a gauge factor of 1.21, suitable for strain sensing.

**Figure 12:**
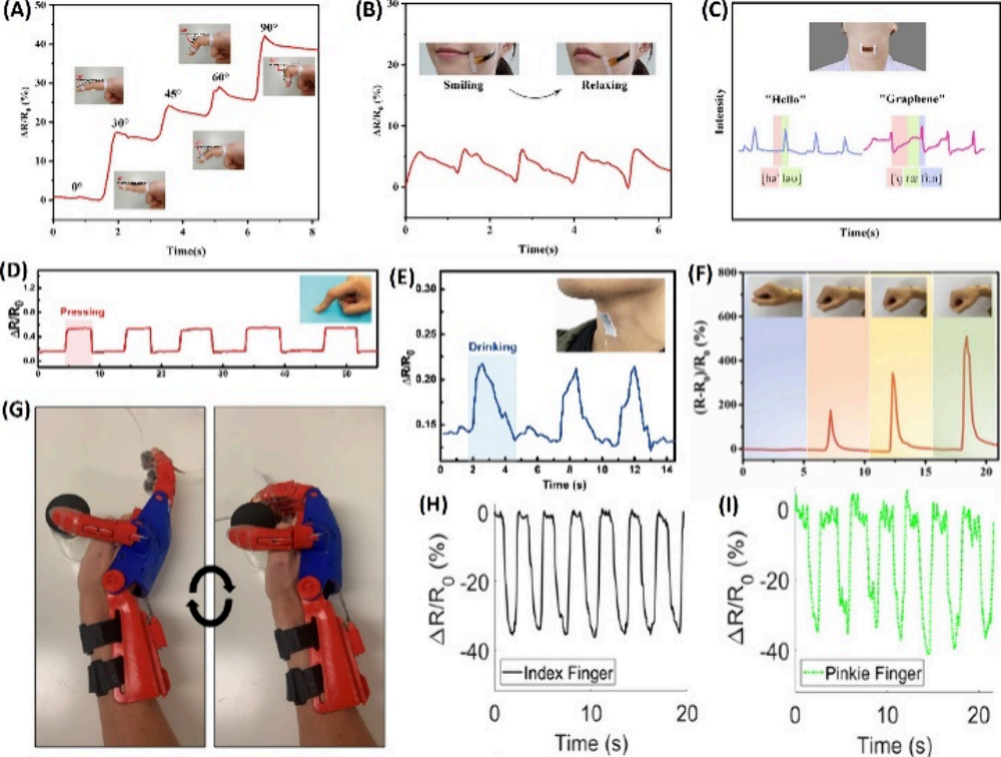
(A–C) Real-time signals detection of human movements, such as finger joint bending, face smiling, and throat vocalizing words. Reprinted with permission from ref (170). Copyright 2022, Elsevier B.V. (D, E) Finger pressing and throat movements while drinking water. Reprinted with permission from ref (171). Copyright 2020, Elsevier B.V. (F) Wrist bending. Reprinted with permission from ref (172). Copyright 2022, Elsevier B.V. (G–I) Fingertip pressure detection on a prosthetic hand that cyclically grasps an object, and respective reading on the index and pinkie fingers. Reprinted with permission from ref (173). Copyright 2021, MDPI.

Wang et al.^170^ reported a novel flexible strain sensor based on a conductive elastomeric PDMS@rGO composite produced for the first time by latex film formation. They achieved the formation of a 3D conductive network with an ultralow amount of rGO, 0.44 vol %, allowing for more mechanically robust and flexible composites. Due to the elastic behavior of the matrix, the improved destruction and reconstruction process of the conductive network under stimuli provided the sensor with excellent sensitivity. Regarding the results, the achieved gauge factor was 6.52 for strain range within 100%, 14.67 for 100–180%, and 24.64 for 180–230%, reaching a peak value of 44.01 at 230–300% strain. The sensor also showed good stability for 2500 load/unload cycles. The resistance changed under strain at a frequency of 0.025 Hz, with cyclic stretching and releasing of the sensor under 10–120% strain resulting in proportional and linear increments of relative resistance variation, an indicator that the sensor had the ability to distinguish different applied strains. The response and recovery times were 165 and 248 ms, respectively, at 100% strain, along with successful monitoring of physiological signals from the human body, detecting subtle human movements such as finger bending (Figure 12A), facial expression changes (Figure 12B), vocal cord vibration, pulse, and speaking (Figure 12C). The chosen fabrication method was generic, scalable, environmentally friendly, and cost-effective.

In a report by He et al.,^171^ a breathable, durable, sensitive, and wearable piezoresistive strain sensor based on a hierarchical microporous PU@CNT composite film was fabricated by the NIPS method followed by dip-coating for deglutition, speech, and human motion monitoring applications. The produced sensors were approximately 8 times more permeable to both air and moisture and the hysteresis related to the operation of these sensors was optimized up to 67.8%, while the structure was stable and durable for more than 8000 cycles. On the other hand, the fabrication method was relatively simple, controllable, and required low energy. In terms of specific parameter combinations, the air permeability of the 10% PU@CNT composite film was 867.76% higher than that of the original nonporous PU films, while its moisture permeability was 801.48% higher than that of the nonporous film. It should be noted that the moisture permeability did not affect the piezoresistive response of the sensor. As the CNT concentration increased, the overall electrical resistance of the composite film decreased, with the maximum sensitivity reaching a value of 51.53 kPa^–1^, at 3 kPa. To test the devices’ performance in long-term e-skin applications, the sensor was successfully used to detect pulse vibrations, finger movements including bending, pressing (Figure 12D), and grasping, and throat movements during vocalization and deglutition (Figure 12E).

Mu et al.^172^ presented a strain/pressure sensor fabricated on the basis of a PDMS@carbon nanocapsule (CNC) composite material through a NaCl-based template method, with the aim of combining high sensitivity with a wide detection range by producing a multilevel porous structure, for human motion monitoring and intrusion detection applications. By combining the excellent electromechanical properties of CNC with the flexibility and toughness of PDMS, the developed sensor exhibited a positive resistance change under a pressure detection range from 0–450 kPa, a maximum gauge factor of 150.7, and stability for at least 2000 load/unload cycles, along with detection occurring in the 0–20% strain range. Additionally, the sensor was able to operate without interfering with the normal daily activities of the human body. The flexible composite film displayed excellent mechanical response to bending, stretching, and twisting motions, along with high elongation at break in the range of 82–124% and a Young’s modulus of up to 110 kPa. By adjusting the ratio of CNC and NaCl template, the conductivity and sensitivity of the film can be tuned, as the hollow structure inherent in CNC provided superior mechanical behavior by acting as a stress-absorbing structure. The sensor could detect finger and wrist (Figure 12F) bending motion without interfering with the user’s activities, and its structural design allowed for a flexible sensor with a wider range while maintaining high sensitivity.

In the work of Herren et al.,^173^ a strain/pressure sponge sensor was fabricated by dispersing CNT in a PDMS matrix, allied with a novel microwave-based porogen removal technique capable of obtaining porous sensors with tunable porosity, piezoresistivity, and electromechanical properties, for human motion monitoring applications. Although all CNT loadings showed similar piezoresistive performance, sensors with CNT at 3 wt % exhibited the highest average sensitivity, along with a gauge factor of 4.8 and a minimum compressive strain detection of 2%, the highest among the samples. This CNT loading displayed the most extensive conductive network, an essential factor that contributed to a more reliable connection with the electrodes. On the other hand, the lower-porosity sensors showed long-term durability and minimal strain rate influence, while exhibiting greater energy absorption and recovery than more porous sensors in the 5–50% strain range. In the 10–5% strain range, the reported gauge factor was comparable to those of other nanocomposite-based sensors. Taking this into account, it was concluded that high-sensitivity and low-porosity sensors have the necessary characteristics for skin-attachable sensors in dynamic human motion detection and monitoring applications. As a result, both footstep detection and compression measurements in a prosthetic hand (Figure 12G–I), as well as sensors attached to elbows, knees, and chest for breathing, walking, running, joint flexion, and throwing motion monitoring, were successfully performed.

Chen et al.^174^ produced a flexible microfluidic sensor based on a wavy-shaped microchannel Ecoflex substrate injected with liquid metal eutectic gallium indium (EGaIn), by a screen-printing technique, for human body and robot joint motion monitoring applications and human respiratory frequency detection. The minimum hysteresis recorded was 1.02% in the working range of 0–320% strain, where the resistance of EGaIn increased 15.72 times, and a gauge factor of 4.91 was recorded at a strain of 320%. The highest recorded resolution was 0.09% strain, along with a resistance response overshoot of 1.9% at 250% strain, in a hold state, a response time of 116 ms, stability during dynamic loading, and long-term stability due to a recorded resistance response overshoot of 1.1% after a 5 day period of static load application.

Althagafy et al.^175^ presented a flexible and conductive smart textile based on a cotton fabric reinforced with drop-cast polyaniline (PANI) + PEDOT:PSS for pressure-sensing applications. The fabricated sensors displayed a minimum sheet resistance of 0.714 kΩ/sq at a PANI concentration of 49.24 wt %, a value 90% lower than the reference sample, along with a conductivity strongly affected by temperature, with metallic behavior observed between 20 and 70 °C and semiconductor behavior between 70 and 160 °C. As the PANI concentration increased, the degree of crystallinity decreased, indicating an increase in conductivity. Finally, this sensor was able to detect changes in pressure, where the resistance varied between 0.438 and 0.429 kΩ, returning to the original value once the application of pressure ceased, showing good stability.

Jin et al.^176^ fabricated a high-performance strain sensor based on Lycra cotton modified with dip-coated polydopamine (PDA) and reinforced with dip-coated rGO and polymerized PANI for human motion tracking, human–machine interface, and medical monitoring applications. While the pristine Lycra cotton showed a maximum strain of 175% and a stress of 5.5 MPa, the fabric exhibited a maximum elongation of 145% under similar stress with deposited rGO, while the completely reinforced fabric had an elongation at break of 50%, with visible fractures above 30% tensile strain. In terms of sensitivity, the sensor showed a gauge factor of 24 in the 0–5% strain range, 6 in the 5–20% strain range, and 1.27 at 20–50% strain range. The sensor captured subtle movements in the 0.2–0.4% strain range, with calculated response and recovery times of 85 and 89 ms, respectively. Lastly, the device had excellent stability from 5% to 40% strain rate up to 1500 cycles. The sensor was used to detect and monitor wrist, knee, finger, and elbow joint motion and demonstrated excellent stability and repeatability.

From the summary presented in Table 4 for these reported flexible strain and pressure sensors, it can be concluded that strain and pressure sensors have high durability and fast response and recovery times, as well as wide-ranging gauge factors, depending on the intended application.

**Table 4:** Featured Published Works for Strain and Pressure Flexible Sensors

key materials	application	main properties	ref
PDMS@MWCNT	human motion monitoring	dry blending + mold casting; percolation threshold reached at ∼2 wt %; gauge factor of 1.21; linear range between 0–40%	(169)
PDMS@rGO	human motion, speech and deglutition monitoring	latex film forming; gauge factor of 44.01; strain up to 300%; response and recovery times 165 and 248 ms	(170)
PU@CNT	deglutition, vocalization and finger motion monitoring	NIPS + dip-coating; durability over 8000 cycles; maximum sensitivity of 51.53 kPa^–1^.	(171)
PDMS@NaCl@carbon nanocapsules (CNC)	human motion monitoring and intruder detection	template method; detection range from 0–450 kPa; gauge factor of 150.7	(172)
PDMS@CNT	human motion detection	solvent-based sonication method; gauge factor of 4.8; minimum compressive strain detection of 2%	(173)
Ecoflex@EGaIn	human body and robot joint movement monitoring; respiratory frequency monitoring	spin-coating; gauge factor of 4.91; hysteresis of 1.02%; working range of 0–320% strain; resolution of 0.09% strain; response time of 116 ms	(174)
cotton@PANI+PEDOT:PSS	pressure sensing	drop-casting; minimum sheet resistance of 0.714 kΩ/sq; semiconductor behavior above 70 °C; resistance variation between 0.438 and 0.429 kΩ when pressure was applied	(175)
Lycra cotton@PDA+rGO+PANI	human motion, human–machine interface, and medical device monitoring	dip-coating + *in situ* polymerization; elongation at break of 50%; maximum gauge factor of 24; Stability up to 1500 cycles; response and recovery times of 85 and 89 ms	(176)

### Sweat Sensors

**Figure 13:**
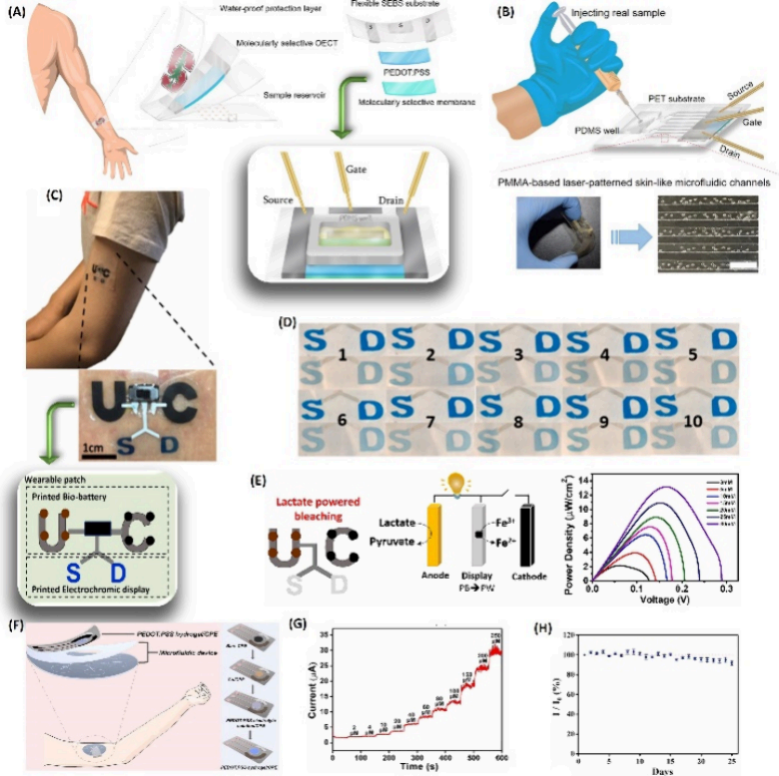
(A) Schematic drawing of the integrated wearable cortisol sensor, with detail of the several important layers: a sweat acquisition channel array, a sample reservoir, a sensor layer, and a protective layer, and an inset of the MS-OECT device. (B) Schematic drawing of skin-like microfluidic device with a photograph of the device being flexed, and an optical micrograph of its surface. Reprinted with permission from ref (177). Copyright 2018, Science Advances. (C) Photograph of wearing the biobattery integrated electrochromic patch, and illustration of the integrated wearable electrochromic patch, including the printed biobattery and electrochromic display. (D) The reversible electrochromic color change performance during ten consecutive coloring/bleaching cycles. (E) Schematic illustration of the lactate-powered electrochromic bleaching, along with the operating principle, and obtained power density in the presence of various lactate concentrations. Reprinted with permission from ref (179). Copyright 2022, Elsevier B.V. (F) Schematic representation of the microfluidic device integrated with the PEDOT:PSS hydrogel/CPE, and the preparation process of the PEDOT:PSS hydrogel-based sensing platform. (G) Amperometric responses of the PEDOT:PSS/CPE to various concentrations of uric acid with an applied potential of 0.4 V. (H) Responses of the UA sensor to 0.2 mM UA recorded every day. Reprinted with permission from ref (181). Copyright 2021, Elsevier B.V.

Parlak et al.^177^ presented an electrochemical transistor that was integrated into a synthetic and biomimetic SEBS@PEDOT:PSS polymeric membrane manufactured by a spin-coating method, which acted as a molecular memory layer for stable and selective detection of cortisol levels, the human stress hormone (Figure 13A). The device was fabricated on a SEBS elastomer substrate, which allowed for a wearable sensor with superior flexibility and stretchability, enabling more precise sample acquisition and delivery to the sensor interface. In addition, a set of laser-patterned microcapillary channels was fabricated as a passive means of flow control (Figure 13B). The system was successfully used in both *ex situ* skin-like microfluidics and on human subjects with real sample analysis. The molecularly selective device exhibited high physicochemical stability at body temperature, along with resistance to induced physical deformation when applied under expected and tolerated conditions, similar to those found in the normal range of motion of the human epidermis. The laser-patterned sweat channels and reservoirs were able to absorb and collect sweat, providing stable and reliable readings while preventing direct mechanical contact between the skin and the sensor.

Payne et al.^178^ developed, optimized, and characterized an enzyme-based amperometric sensor for lactate-level monitoring applications. Lactate oxidase was chosen as the sensing mechanism, while TTF acted as a mediator, achieving results optimized for athletic applications. The sensor showed a linear range up to 24 mM lactate and a sensitivity of 68 μA cm^–2^/mM, taking into account the surface area of the sensor. It was observed that sodium chloride (NaCl) and potassium chloride (KCl) concentrations decreased the enzyme activity and the resulting electrical current, thus affecting the performance of the sensor. Increases in CaCl_2_ concentration induced nonlinear variations in sensor performance and enzyme activity. In the produced sensor, a change in lactate corresponded to a change in current of ∼10 μA. When measuring the current changes induced by salt perturbation, it was observed that the values were below the threshold required to distinguish between aerobic and anaerobic metabolism.

For these reasons, while the advantages of this sensor were relevant, the accuracy of blood and spectroscopic sweat tests cannot be surpassed by this device. However, since only variations in lactate concentration affected the enzyme activity in the sensor, the sensor manages to circumvent possible interferences caused by other electrochemical reactions, functioning as a good indicator of the transition between aerobic and anaerobic metabolism. According to the authors, it is unlikely that enzymatic lactate sensors will be able to operate in continuous health monitoring applications.

Hartel et al.^179^ reported a fully screen-printed ion-selective electrode and electrochromic self-powered sweat sensor for sweat lactate monitoring applications (Figure 13C). The device exhibited reversible and strong color changes over ten cycles, depending on concentration and time profiles (Figure 13D). Further results showed that the device had potential for continuous on-skin lactate level monitoring with easy reversibility, ease of fabrication, and simple colorimetric readouts. The sensor operated and adapted to different analyte concentrations, covering the entire physiological range, with power generation up to 13 μW/cm^2^, twice the amount needed to successfully bleach the electrochromic display within 2 min (Figure 13E). These results, including self-generation of power from sweat, ease of data readout, mass reduction potential, and stable reversible electrochromic performance, demonstrated the device’s potential for practical applications, with possible extension to other enzymatic sensing systems, such as ethanol monitoring to promote safer driving or glucose monitoring for diabetic patients.

Zheng et al.^180^ proposed a flexible and wearable fabric-based sweat sensor developed by multilevel screen-printing and drop coating for glucose level analysis applications. The produced sensor exhibited excellent sweat collection and transport channels actuated by a microchannel network provided on the bottom side of the sensor, which was helpful to collect flowing sweat on the skin surface, along with shortened sweat collection times due to the use of capillary forces to efficiently absorb the biofluid, a simple and cost-effective approach achieved by today’s screen-printing technology. Under optimal conditions, the sweat sensor could measure the concentration of sweat glucose in the range of 0.05–1 mM with a sensitivity of 105.93 μA/(mM cm) for up to 9 h, along with excellent selectivity, reproducibility, and stability. The sensor’s performance was consistent with that of glucose kits. It was concluded that the sweat collection and transport channels could provide durable and stable detection of glucose in sweat, that screen-printing and drop-coating methods could easily produce sensors at a low cost, and that the sensor was skin-friendly and durable because the working electrode avoided direct contact with the skin. In addition, the sensor exhibited acceptable detection range, sensitivity, reproducibility, stability, and selectivity under optimal conditions.

Xu et al.^181^ reported a wearable microfluidic-based amperometric sweat sensor manufactured by incorporating PEDOT:PSS and copper in a PDMS matrix for uric acid level monitoring applications (Figure 13F). The PDMS microfluidic device was responsible for real-time sweat sensing, while the conductive and large-area PEDOT:PSS enhanced the overall flexibility and detected uric acid levels and stored electrolytes. The sensor achieved an ultrahigh sensitivity of 0.875 μA/(μM cm) and a low limit of detection of 1.2 μM. Compared with other uric acid sensors, this sensor had a much lower detection limit and a suitable linear range between 30 and 80 μM, in the range of 2–250 μM for uric acid detection in human sweat. It was believed that this sensor had potential for high-performance monitoring of biomarkers, metabolites, and nutrients. The porous structure and large specific surface area of PEDOT:PSS provided the composite with excellent electrochemical behavior, with the largest recorded response to uric acid levels observed at an applied potential of 0.4 V, along with acceptable levels of background noise (Figure 13G). Furthermore, the output signal remained constant for 50 cycles, demonstrating electrochemical stability, along with long-term stability, as the sensor was also tested across 25 days, retaining more than 95% of the initial signal response to uric acid (Figure 13H). The sensor’s performance was not affected by the presence of other substances such as ascorbic acid, lactic acid, glucose, or ethanol, whose concentrations are commonly significant in sweat, demonstrating the selectivity of the device. Finally, it took 166 s to completely fill the microfluidic sweat reservoir, while repeated induced deformations did not significantly affect the electrochemical performance of the sensor, both promising characteristics for wearable sensors that can adapt to the user’s daily life.

Lv et al.^182^ reported a flexible ammonia gas sensor based on a porous PVDF substrate and a poly(aniline-*co*-pyrrole) active film prepared by *in situ* polymerization, for ammonia concentration detection in human body applications. The sensor exhibited excellent sensing performance, detecting the presence of NH_3_ at a minimum concentration of 0.05 ppm with a response value of 6.7% at room temperature and 70% RH. At a concentration of 20 ppm, the maximum response was 1368%. The response time and recovery time of the sensor were 136 and 167 s, respectively, while the response value of the device at a concentration of 1 ppm decreased from 93% to 79% after 6000 bend/extend cycles and to 75% after 8000 cycles. When the temperature was increased from 25 to 65 °C, the response value decreased to 55%, with a resistance change from 535 Ω to 2 kΩ. The response of the sensing film increased up to 85% RH and decreased significantly at 95% RH. Thus, the sensor exhibited good ammonia response, selectivity, stability, repeatability, and good flexibility.

Wang et al.^183^ developed a photo-cross-linked flexible SERS wearable sensor based on sulfonated cellulose nanofibers (S-CNF) reinforced with AgNP and acrylic acid (AA), resulting in a nanocomposite hydrogel (S-CNF-Ag NPs/PAA), for urea, uric acid, and pH level monitoring applications. The manufactured sensor exhibited stress and strain values up to 1209 kPa and 612%, respectively, and good adhesion properties with an interfacial toughness of ∼116.23 J/m^2^. Regarding stability, the sensor displayed excellent SERS behavior after withstanding up to 1200 cycles of stretching and kneading. When used to monitor urea and uric acid, the linear ranges were 0.1–1000 and 0.005–1 mM, respectively, with good correlation coefficients of *R*^2^ = 0.9984 and *R*^2^ = 0.9865 and detection limits of 63.1 and 3.98 μM, respectively. In the case of pH level monitoring, the sensor showed high sensitivity ranging from 5.80 to 7.60, which is similar to the performance and results of a commercial pH meter and very similar to adult sweat pH values ranging from 5.5 to 7.0. These experimental results denote a high degree of practicality for human skin sweat analysis and pH detection, while also showing antimicrobial properties, where the bacterial inhibition rate reached a maximum value of 93%, due to disruption of metabolic processes and induction of bacterial cell damage and death.

Li et al.^184^ reported a flexible hydrogel-based sweat and human motion sensor composed of PVA modified with cellulose nanocrystals reinforced with PDA and AuNPs, for lactate level and human motion monitoring applications. The prepared sensors exhibited maximum stress values ranging from 0.40 up to 0.80 MPa, which increased with the reinforcement content. The self-healing efficiency of the hydrogel reached a maximum of 87.6%, with razor blade nicks disappearing after 6 h. The sensors showed stability up to 10 tensile cycles in the 0–100% strain range. In terms of sensitivity under mechanical deformation, a maximum gauge factor of 0.99 was observed in the strain range of 0–350%, with a response time as low as 160 ms, along with stable curves, indicating sensor stability. When used to monitor lactate levels, the lactate oxidase (LOx) enzyme was affected by temperature, reaching maximum performance at 35 °C and decreasing above 40 °C, and a pH level of 7.0 was considered optimal for the LOx biological activity, both levels found in the human body. Moreover, a sensitivity of 98.0 nA/mM was recorded together with a linearity of *R*^2^ = 0.9987 in the linear range of 0.5–30 mM, and a detection threshold of 0.31 mM, values very similar to the lactate concentration in human sweat (1–20 mM). Therefore, it can be assumed that this sensor can reliably monitor joint motion along with lactate concentration in human sweat for future healthcare applications.

Table 5 shows the featured published works regarding flexible sweat sensors. From the information reported it is observed that the trend in this field is the production of sensors capable of stable, reliable, and continuous operation, with great linearity and sensitivity, without sacrificing the mechanical properties.

**Table 5:** Featured Published Works for Flexible Sweat Sensors

key materials	application	main properties	ref
SEBS@PEDOT:PSS+Ag/AgCl	cortisol-level monitoring	laser-patterning + spin-coating; superior flexibility and stretchability; high physicochemical stability; stable and reliable readings	(177)
Lactate oxidase+ TTF+ chitosan/CNT; Ag/AgCl; Gold	lactate-level monitoring	inkjet printing + screen-printing; linear range up to 24 mM lactate; sensitivity of 68 μA cm^–2^/mM; NaCl and KCl concentrations decreased enzyme activity	(178)
PET@Carbon Ink; Lactate oxidase; TTF+MWCNT; Prussian Blue mediator	biofuel cells and lactate-level monitoring	screen-printing; color changes for over ten cycles; continuous lactate-level monitoring; power generation up to 13 μW/cm^2^	(179)
Cloth/Paper@Carbon Ink; GOx; Prussian Blue + MWCNT mediator	glucose-level monitoring	screen printing + drop coating; glucose measured in the 0.05–1 mM range; sensitivity of 105.93 μA/(mM cm) up to 9 h	(180)
PDMS@PEDOT:PSS+Cu	uric acid-level monitoring	etching + electrodeposition + spin-coating; sensitivity of 0.875 μA/(μM cm); low detection threshold of 1.2 μM; linear range between 2–250 μM; 95% of the signal response remained after 25 days of testing	(181)
PVDF@poly(aniline-*co*-pyrrole)	ammonia concentration detection	*in situ* polymerization; detection at a minimum concentration of 0.05 ppm; maximum response value of 1368%; response and recovery times of 136 and 167 s; sability up to 8000 cycles	(182)
S-CNF@AgNP+AA	urea, uric acid, and pH level monitoring	photo-cross-linking; urea and uric acid detection in the 0.1–1000 and 0.005–1 mM ranges; detection thresholds of 63.1 and 3.98 μM; high sensitivity in the 5.80–7.60 pH level range; stability up to 1200 cycles; bacterial inhibition rate of 93%	(183)
PVA@cellulose nanocrystals+PDA+AuNPs	lactate level and human motion monitoring	*in situ* polymerization+freeze–thaw; maximum stress of 0.8 MPa; maximum gauge factor of 0.99; maximum biological activity at 35 °C and 7.0 pH; response time of 160 ms; lactate sensitivity of 98.0 nA/mM; detection threshold of 0.31 mM; linear range of 0.5–30 mM	(184)

## Conclusions and Final Remarks

Throughout this work, the most used flexible and wearable sensors have been described in detail, with an additional focus on their required properties, along with the most chosen materials, composites, and the interactions between their elements. In addition, the requirements for material selection have also been addressed, indicating their importance and influence on the overall performance of the sensor. The development of a sensor system is a complex task that requires multidisciplinary efforts and the combination of different types of parameters to achieve the desired and competitive sensing performance. Regarding the different types of sensors addressed in this review, the desired properties are common to all of them, namely high flexibility, competitive sensitivity over a wide sensing range, low hysteresis, linearity, durability, and stability, along with low response and recovery times. On the other hand, the produced sensors must achieve the necessary mechanical compliance with the human skin, in order to better adhere to it and mimic its functions.

For these reasons, carbon-based composites are popular in research because of their electromechanical stability, low cost, high variety, and known behavior. Among them, graphene, although a relatively new material, has attracted great interest due to its excellent electromechanical behavior, high Young’s modulus, exceptional specific surface area, and suitable electrical conductivity.

In conclusion, the available and applied fabrication methods for each type of sensor differ significantly, as well as the commonly chosen materials. Additionally, both the functionalization and the manufacturing method, as well as the weight ratio used between the matrix and the electrically conductive reinforcement, have a significant influence on the results obtained at the end of the process, since nowadays there are a large number of possible composite combinations for the same application in sensor systems. Lastly, research effort trends focus on manufacturing methods that lead to simpler, less expensive, scalable, and environmentally friendly processes, characteristics that are crucial for encouraging the commercialization of the technology in new, more individualized devices and compatibility with faster rehabilitation processes.
